# Time to Recurrence and Survival in Serous Ovarian Tumors Predicted from Integrated Genomic Profiles

**DOI:** 10.1371/journal.pone.0024709

**Published:** 2011-11-03

**Authors:** Parminder K. Mankoo, Ronglai Shen, Nikolaus Schultz, Douglas A. Levine, Chris Sander

**Affiliations:** 1 Computational Biology Center, Memorial Sloan-Kettering Cancer Center, New York, New York, United States of America; 2 Department of Epidemiology and Biostatistics, Memorial Sloan-Kettering Cancer Center, New York, New York, United States of America; 3 Gynecology Service, Department of Surgery, Memorial Sloan-Kettering Cancer Center, New York, New York, United States of America; Virginia Commonwealth University, United States of America

## Abstract

**Background:**

Serous ovarian cancer (SeOvCa) is an aggressive disease with differential and often inadequate therapeutic outcome after standard treatment. The Cancer Genome Atlas (TCGA) has provided rich molecular and genetic profiles from hundreds of primary surgical samples. These profiles confirm mutations of TP53 in ∼100% of patients and an extraordinarily complex profile of DNA copy number changes with considerable patient-to-patient diversity. This raises the joint challenge of exploiting all new available datasets and reducing their confounding complexity for the purpose of predicting clinical outcomes and identifying disease relevant pathway alterations. We therefore set out to use multi-data type genomic profiles (mRNA, DNA methylation, DNA copy-number alteration and microRNA) available from TCGA to identify prognostic signatures for the prediction of progression-free survival (PFS) and overall survival (OS).

**Methodology/Principal Findings:**

We implemented a multivariate Cox Lasso model and median *time-to-event* prediction algorithm and applied it to two datasets integrated from the four genomic data types. We (1) selected features through cross-validation; (2) generated a prognostic index for patient risk stratification; and (3) directly predicted continuous clinical outcome measures, that is, the time to recurrence and survival time. We used Kaplan-Meier p-values, hazard ratios (HR), and concordance probability estimates (CPE) to assess prediction performance, comparing separate and integrated datasets. Data integration resulted in the best PFS signature (withheld data: p-value = 0.008; HR = 2.83; CPE = 0.72).

**Conclusions/Significance:**

We provide a prediction tool that inputs genomic profiles of primary surgical samples and generates patient-specific predictions for the time to recurrence and survival, along with outcome risk predictions. Using integrated genomic profiles resulted in information gain for prediction of outcomes. Pathway analysis provided potential insights into functional changes affecting disease progression. The prognostic signatures, if prospectively validated, may be useful for interpreting therapeutic outcomes for clinical trials that aim to improve the therapy for SeOvCa patients.

## Introduction

Ovarian cancer is considered a “silent” disease, as 70% of patients are diagnosed at an advanced stage with high grade serous ovarian cancer (SeOvCa) [1]. The standard treatment requires cytoreduction surgery followed by administration of platinum and taxane-based chemotherapy. In a large number of patients with advanced stage papillary SeOvCa (stages III/IV) that initially respond to primary treatment with surgery and chemotherapy, cancer recurs with a drug-resistant phenotype (25% cases within 6 months) and overall 5-year survival is 31% [1]. Consequently, there is an urgent need for diagnostic molecular features or biomarkers that can be associated with survival and disease recurrence in SeOvCa.

Recently, mRNA expression signatures that predict platinum-resistance [2], progression- free survival [3] or overall survival [2], [4], [5] have been developed. Although these studies provided valuable first clues to molecular changes in SeOvCa that might be exploited in new treatment strategies, most of them suffered from limited sample size, and the number of overlapping genes in the identified profiles was minimal. It is well known that statistically derived signatures are not necessarily unique, possibly because of individual variation, heterogeneity [6] or collinearity [7]. However, given the diversity and extent of copy number alterations in SeOvCa genome, having a large sample size is a prerequisite for accurately identifying alterations that could be most associated with tumor recurrence and patient overall survival. A meta-analysis using nine published gene sets [8] found, among others, oxidative stress response mediated by NRF2, TP53 signaling and TGFβ signaling to be associated with platinum based therapy resistance.

Since clinical decisions are usually binary, methods like support vector machines [9], [10] and univariate Cox regression are typically utilized to stratify patients into binary categories such as *bad*-prognosis and *good*-prognosis (or *low*- and *high*-risk). However, the availability of clinical time data provides an opportunity to directly predict *time-to-event* and can hopefully lead to a more informative signature that can be reduced to binary decisions with no loss of information. Clinical *time-to-event* prediction is possible but is a relatively unexplored field and a challenging task.

The Cancer Genome Atlas (TCGA) project was established to profile large tumor sets at both the DNA and RNA level to create an integrated atlas of the aberrations present in tumor cells. Ovarian Cancer is the second tumor type analyzed by TCGA, and the study focused on newly diagnosed untreated invasive high-grade SeOvCa samples.

Using TCGA data, the aims of our study were to (1) develop molecular signatures of individual data types (mRNA expression, microRNA expression, DNA methylation and copy-number alteration data from primary surgical samples) associated with platinum-free interval, progression-free survival and overall survival in advanced-stage SeOvCa; (2) integrate four different data types and compare the performance of genomic integration with the individual data types; (3) test the predictive power of our signatures in withheld data, and, wherever possible, in other fully independent and publicly available datasets of high-grade SeOvCa; and (4) derive the network of interactions and associated pathways and transcription factors.

To achieve these goals, we utilized the wealth of information available from TCGA and implemented an L_1_-regularized Cox proportional hazards model to do feature selection using the Cox model with an L_1_ penalty as proposed by Park and Hastie [11]. Previously published mRNA expression datasets were used to test our gene signatures created from TCGA mRNA expression data. Further we investigated the network of interactions and associated pathways resulting from our signatures and identified pathways and processes that could possibly explain the biological behavior of SeOvCa.

## Results and Discussion

### Clinical Characteristics of the TCGA Data

Outcome measures of interest for our analysis were overall survival (OS), progression-free survival (PFS) and platinum-free interval (PFI) (Table 1). OS was defined as the time between the initial surgical resection to the date of last follow-up or death. PFS was defined as the interval from the date of initial surgical resection to the date of progression, date of recurrence, or date of last known contact, if the patient was alive and has not recurred. PFI was defined as the interval from the date of last primary platinum treatment to the date of progression, date of recurrence, or date of last known contact if the patient is alive and had not recurred. Tumor recurrence was defined using criteria customary to the contributing institution. Platinum status was defined as resistant if the PFI was less than six months and the tumor had progressed or recurred. Platinum status was defined as sensitive if the platinum free interval was six months or greater, there was no evidence of progression or recurrence, and the follow-up interval was at least six months from the date of last primary platinum treatment [1].

**Table 1:** Clinical characteristics of the TCGA data.

Cohort	OS	PFS/TTP	PFI
Number of patients	481	395	287
Median Age	59.1	58.7	58.7
Serous	481	395	287
Platinum status			
Sensitive	195	195	195
Resistant	92	92	92
Recurrent disease			
No	113	113	40
Yes	282	282	247
Vital status			
Alive	213	194	116
Dead	268	200	170
Median time, months	43.6	16.8	10.4

The total number of TCGA patients available (and associated clinical characteristics) within each clinical outcome measure category are reported. All outcome measures are depicted in the units of months.

**Figure 1:**
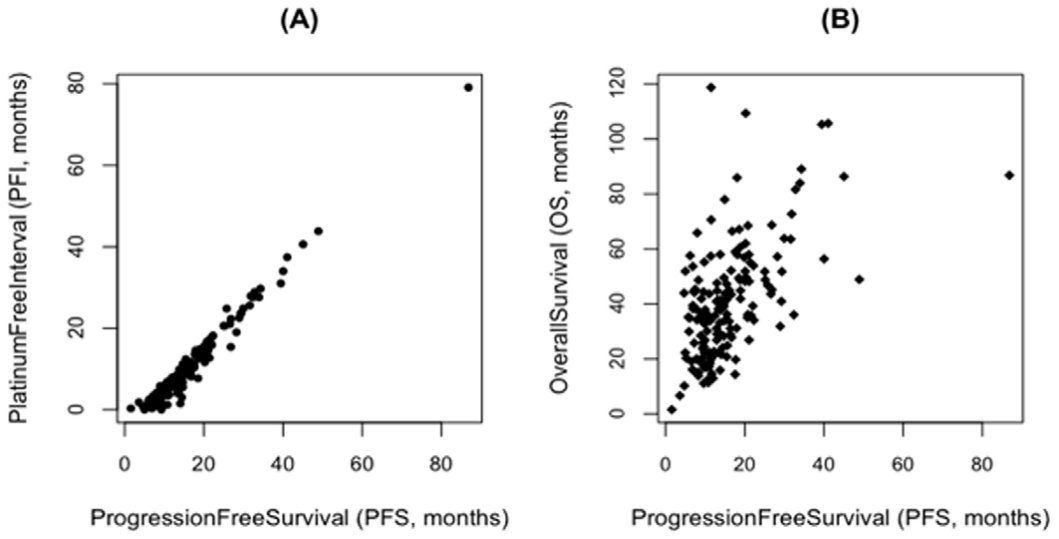
Correlation of TCGA clinical outcome measures. (A) PFS and PFI are strongly correlated and do not need to be predicted separately. (B) PFS and OS are not well correlated, so we derived separate predictive signatures for each (data only for un-censored patients).

PFS and PFI data were only available for a subset of patients (Table 1) and were last updated on September 13, 2010. The PFI and PFS outcome measures were directly correlated with each other (Figure 1A), and PFS and OS outcome measures were not (Figure 1B). Therefore, our approach was to identify different signatures to predict PFS and OS, respectively. We chose to predict PFS rather than PFI for two reasons: 1) there were ∼100 more cases available with PFS information than PFI (potential for improving the statistical performance of molecular signatures identified), and 2) PFS is the outcome measure that is generally reported in the literature.

### Molecular Signature from Individual Data types

To achieve our first goal of deriving molecular signatures from individual data types (mRNA expression, microRNA expression, DNA methylation and copy-number alteration data) most associated with tumor recurrence and survival, we implemented a multivariate Cox Lasso model. This model is a path following algorithm for L_1_-regularized Cox proportional hazards model [11] and reports the markers of outcome through a cross-validation procedure and maximization of concordance probability estimates.

A potential issue in developing predictive signatures is over-fitting to the training dataset, resulting in a signature that reflects the characteristics of the training set but cannot accurately predict outcome in the test set. Consequently, a fairly rigorous cross-validation procedure of the regression model was followed and the models were parameterized during the training procedure and fixed before moving to the test data analysis. For creating the training set, 316/395 cases with PFS data and 384/481 cases with OS data were randomly chosen, and the rest were used as a blind test set of the resulting molecular signatures. Three measures of performance of the signatures for the test data were selected: p-value (the measure of how well the signature stratifies patients into broadly defined health-risk categories), Hazard ratios (HR, the ratio of rate at which patients in two groups are experiencing events), and concordance probability estimates (CPE, a measure of how well our signatures predict the correct order of median *time-to-event*). The cross-validation CPE (cv.CPE) and the CPE of the test data (CPE.test) are provided for each data type. The total number of features resulting from the four individual data types and the respective integrated versions for the two outcome measures are summarized in Table 2.

**Table 2:** Results from individual data types and the integrated versions.

	Progression Free Survival (PFS)					
Data type	features	CPE.test	c-scorep-val	t-scorep-val	HR	95% CI
mRNA	181	0.77	0.17	0.05	1.97	(0.94, 4.11)
microRNA	81	0.63	0.09	NA	1.48	(0.75, 2.91)
DNA Methylation	140	0.72	0.03	NA	1.96	(1.05, 3.63)
Copy Number Alteration	167	0.67	0.61	NA	1.36	(0.71, 2.59)
Integrated data	156	0.72	0.008	0.004	2.83	(1.40, 5.74)

	Overall Survival (OS)					
Data type	features	CPE.test	c-scorep-val	t-scorep-val	HR	95% CI
mRNA	219	0.80	0.70	0.09	1.26	(0.62, 2.55)
microRNA	87	0.69	0.09	NA	1.22	(0.63, 2.35)
DNA Methylation	171	0.74	0.52	NA	1.45	(0.77, 2.75)
Copy Number Alteration	278	0.75	0.18	NA	0.77	(0.39, 1.53)
Integrated data	182	0.73	0.59	0.81	1.10	(0.54, 2.22)

The number of features and four measures of performance are provided for the PFS (top) and OS (bottom) signatures. Hazard Ratio (HR) and 95% confidence interval (CI) are reported for *low*- and *high*-risk groups based on Cox score (c-score) stratification.

#### Copy-number Alteration Data

Given the extent of copy number alterations (CNA) and the relatively low number of significantly mutated genes observed in SeOvCa, it is considered a copy-number driven disease. Consequently, we tried to identify copy-number features most associated with the different outcome measures.

Using our methodology, 167 copy-number features (genes) were found to be most associated with PFS and 278 features most associated with OS. All analysis details are provided in File S4. The CPE.test for recurrence analysis was 0.67 and CPE.test for survival analysis was 0.75 (Figures 4SA, 4SB in File S4). The patient-risk stratification (tertile stratification using c-scores) for the test set was not statistically significant for recurrence and survival data (Table 2).

#### mRNA Expression, microRNA Expression and DNA Methylation Data

**Figure 2:**
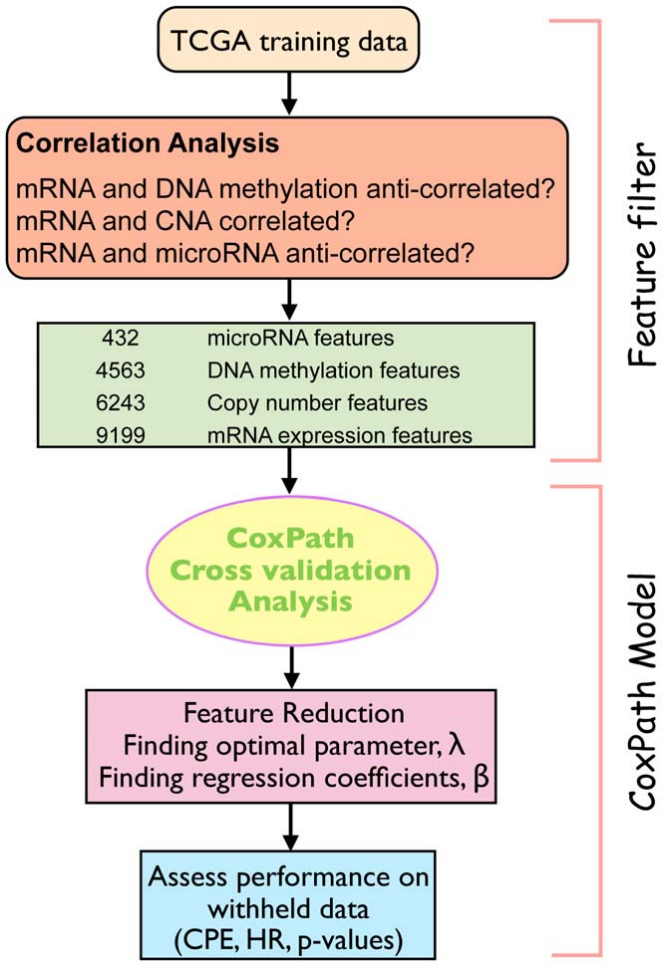
Integration Procedure and CoxPath Methodology. Integration combines multiple data types for the multivariate Cox Proportional hazards model.

**Figure 3:**
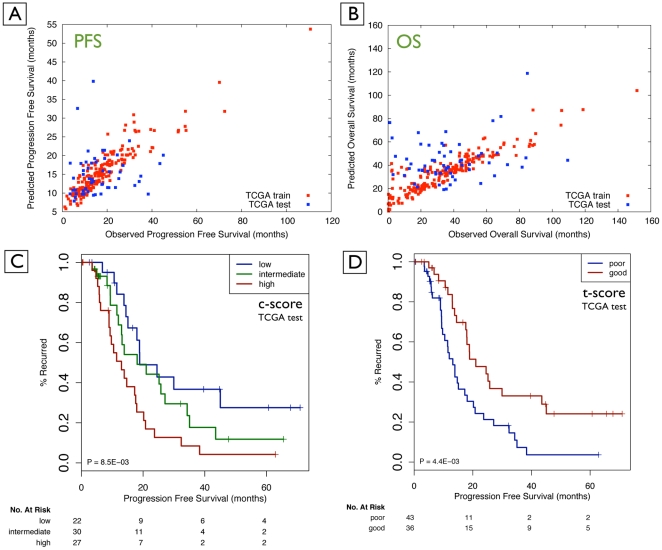
Quality of outcome prediction for survival time (A, B) and discrete risk categories (C, D). (A) Prediction of *time-to-event* (PFS; un-censored data); (B) prediction of *time-to-event* (OS; un-censored data); (C) statistically significant stratification into *low*-, *intermediate*- and *high*-risk patients using the prediction method for TCGA test data based on c-score (Integrated PFS signature); and (D) stratification for the TCGA test data based on t-score (Integrated PFS signature).

The mRNA expression analysis for PFS data identified 181 features that stratified TCGA test data with p-value = 0.05 (t-score) and 0.17 (c-score) and resulted in CPE.test = 0.77 (Figures 1SA, 1SB in File S1). For OS, 219 features were identified resulting in stratification p-value = 0.09 (t-score) and 0.70 (c-score) and CPE.test = 0.80 (Figures 1SC, 1SD in File S1). The DNA methylation analysis for PFS identified 140 features with p-value = 0.03 (c-score, test data) and CPE.test = 0.72. For survival, DNA methylation identified 171 features with p-value = 0.52 (c-score, test data) and CPE.test = 0.74 (Figures 2SA, 2SB in File S2). The microRNA analysis for PFS identified 81 features with p-value = 0.09 (c-score, test data) and CPE.test = 0.63. For survival, microRNA analysis identified 87 features with p-value = 0.09 (c-score, test data) and CPE.test = 0.69 (Figures 3SA, 3SB in File S3).

The details of the data processing, the methodology and results from the three data types (mRNA expression, microRNA expression and DNA methylation) are provided in Files S1, S2 and S3, respectively.

Based on the c-scores performance of the individual data types, the DNA methylation signature is the most statistically significant, the microRNA signature is borderline significant among PFS signatures, and microRNA is borderline significant among OS signatures (Table 2). The molecular signatures based on the TCGA data were also tested in some external datasets (Table 3; Figures 1SA, 1SB, 1SC, 1SD in File S1). This indicated the robustness of the mRNA signatures and their broad applicability.

**Table 3:** Results for the mRNA prognostic signature applied to external datasets.

Data type	features	CPE.test	c-scorep-val	t-scorep-val	HR	95% CI
Tothill (OS)	219	0.80	0.047	0.014	2.06	(1.11, 3.30)
Dressman (OS)	219	0.78	0.008	0.033	1.33	(0.61, 2.88)
Bonome (OS)	219	0.75	0.049	0.180	1.77	(1.09, 2.88)
Tothill (PFS)	181	0.77	0.035	0.012	1.73	(1.10, 2.71)
Bonome (PFS)	181	0.77	0.870	0.880	1.06	(0.68, 1.66)

The number of features and four measures of performance are provided for the PFS and OS mRNA signatures. Hazard Ratio (HR) and 95% confidence interval (CI) are reported for *low*- and *high*-risk groups based on Cox scores (c-score) stratification.

### Molecular Signatures from Integrated Data

Causality is not necessarily established by a correlation between a set of genes and clinical endpoints. Various mechanisms that regulate gene expression include DNA methylation, histone deacetylation, copy-number changes and targeting by microRNAs. Therefore, an integration procedure that incorporates this biologically useful knowledge and is computationally efficient was highly desirable. We created a vector space integration method, which is described in the Methods and Materials sections (Figure 2). This methodology allowed us to directly compare the performance of the integrated method with the performance of the individual data types.

#### Progression-Free Survival

The result of the multivariate Cox Lasso model using the integrated data was 156 features, comprising 85 mRNA features, 47 DNA methylation features, 18 copy-number features, and 6 microRNA features that emerged as being most associated with tumor recurrence. Tertile stratification was performed on the training data. The resulting thresholds for stratifying patients into *low*-, *intermediate*- and *high*-risk groups resulted in a p-value of 8.0e-03 (c-score) and 4.0e-03 (t-score) for the test data (Table 2, Figure 3). The concordance probability of the test data (CPE.test) was 0.72 and HR between *low*- and *high*-risk groups was 2.83. These results suggest an excellent predictive power of the features for patient-risk stratification and median *time-to-event* predictions for tumor recurrence.

#### Overall Survival

The multivariate Cox Lasso model using integrated data resulted in 182 features that were most associated with overall survival. This signature was comprised of 102 mRNA features, 40 DNA methylation features, 30 copy-number features, and 10 microRNA features. Tertile stratification was performed on the training data. The resulting thresholds for stratifying patients into *low*-, *intermediate*- and *high*-risk groups led to a p-value of 0.59 (c-score) and 0.81 (t-score) for the test data. The CPE.test was 0.73 (Figure 3). These results show inferior performance of the integrated survival signature compared to the integrated PFS signature. The median *time-to-event* prediction for the follow-up times (PFS and OS) from the integrated datasets is provided in File S5 (Figure 5SA).

Since the PFS signature identified from genomic integration had the overall best performance (p = 0.008; HR = 2.83; CPE = 0.72), we limit the subsequent gene-set analysis and subsequent network and pathway analysis to the PFS integrated signature.

### Pathway Analysis of the PFS Signature Identified from Genomic Integration

To identify common biological pathways and known interactions of the 156 features in the integrated PFS signature, we applied two different approaches: A) General over-representation analysis to identify over-represented pathways and gene ontology categories, and B) Network analysis to identify genes with evidence for physical or functional interactions (connected in protein-protein interaction or transcriptional networks).

#### A. Over-representation Analysis of the 156-feature Gene Signature

**Figure 4:**
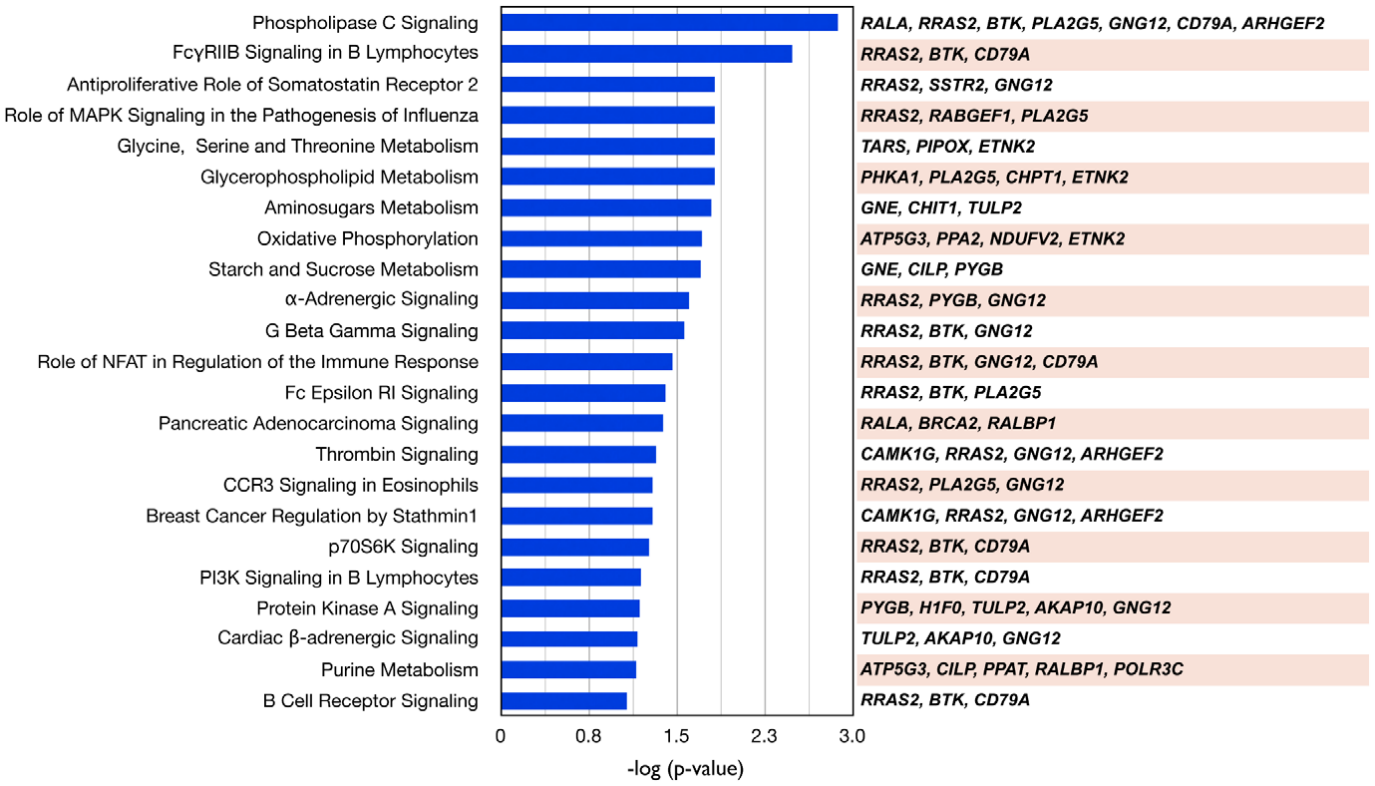
Canonical pathway analysis of 156 genes from the integrated PFS gene signature. IPA [12] identified 23 statistically significant canonical pathways (p<0.1 and ≥3 genes).

Firstly, functional categories from IPA pathways (Ingenuity, Inc.) [12] were used to identify pathways (more precisely, gene sets grouped in pathways) over-represented in the integrated PFS gene signature. Significant pathways (15 pathways with p<0.05 and 23 pathways with p<0.1) containing more than two genes in the signature included phospholipase C signaling, FcγRIIB signaling in B lymphocytes, anti-proliferative role of somatostatin receptor 2, G beta gamma signaling, oxidative phosphorylation, breast cancer regulation by stathmin1, pancreatic adenocarcinoma signaling, α-adrenergic signaling and others (Figure 4). Interestingly, several genes are common to several of the IPA gene sets, such as RRAS2, BTK, CD79A and GNG12.

Secondly, IPA was also used to investigate the biological functions and/or disease association of genes in the PFS signature (31 categories with p<0.05 and genes ≥3 genes). These over-represented categories included cell death & cell cycle; cancer; DNA replication, recombination & repair; cell-to-cell signaling and interaction; metabolic disease; drug & lipid metabolism; inflammatory response; molecular transport, reproductive system development & function; immune system trafficking, tumor morphology; and cellular growth & proliferation and others (Table S1).

**Figure 5:**
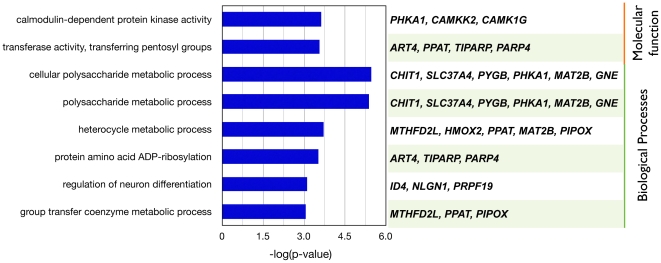
Overrepresented GO categories for genes in the integrated PFS signature. Six biological processes categories and two molecular function categories were indentified by Bingo [13] containing (3<n<100) genes in the signature, a corrected p-value of <0.1.

In addition, to determine which Gene Ontology (GO) categories are statistically overrepresented in the gene signature, we use the Bingo software [13], which is available as a plugin in Cytoscape [14]. Eight GO categories (biological processes and molecular function) were enriched among the 156 features. They were calmodulin-dependent protein kinase activity and transferase activity, polysaccharide metabolic process, protein amino acid ADP-ribosylation and group transfer coenzyme metabolic process (corrected p-value<0.1; Figure 5). We have not further investigated the details of these functions potentially associated with tumor biology, but they represent a guide to further analysis and, possibly, experiments.

#### B. Network Analysis of the 156-feature Gene Signature

In order to investigate functional (sub)networks involving genes in the PFS signature we applied Ingenuity Pathways Analysis [12] and the network analysis algorithm Netbox [15].

**Figure 6:**
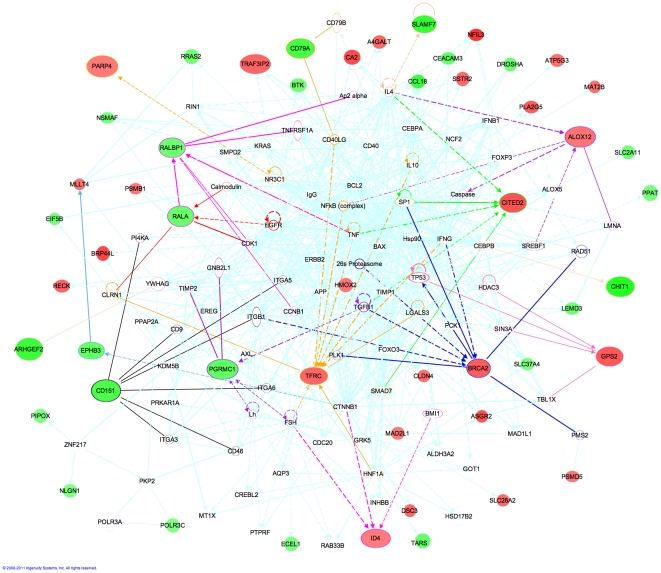
Network derived from the integrated PFS signature using IPA. The top four networks identified were merged using IPA analysis. The features most discriminative between short and long-recurrence times are shown on larger scale. The nearest neighbor interactions of these nodes are highlighted in different colors. Nodes are colored based on the mRNA expression profile of different genes (green: down-regulated in short recurrence patients (PFS<6mo) compared to long recurrence (PFS>40mo), and red: up-regulated).

Firstly, an IPA “Core Analysis” was used (graphical representation) revealing four functional networks: cellular growth and proliferation; hematological system development and function, humoral immune response (network-1); cell-to-cell signaling and interaction, tissue development, cellular movement (network-2); cell cycle, cell death and cancer (network-3); and, cancer, gastrointestinal disease, genetic disorder (network-4). Given the overlap between the networks (TP53, FOXO3, NCF2, SIN3A, CCNB1), we merged the four networks into a single network using the IPA “Merge Networks” tool (Figure 6).

We next tried to identify the genes in this network that are most discriminative between short and long-recurrence times. We observed that BRCA2 (Breast Cancer 2 susceptibility protein; mRNA) is expressed at higher levels in patients with shorter recurrence time. Consistent with this observation, up-regulation of BRCA2 (and BRCA1) has been observed in rapidly proliferating and differentiating cells [16], and following exposure to cisplatin, the DNA repair genes BRCA2 and FANCA have been observed to be up-regulated [17]. Many genes in our signature are, directly or *in*-directly, connected with TP53 (tumor protein p53). GPS2 (G protein pathway suppressor 2) and BRCA2 have the largest expression differences between patients with long and short recurrence times. Interestingly, both of these genes are mutated in SeOvCa, TP53 in 96.5% and BRCA2 in 9.2% of patients [1].

**Figure 7:**
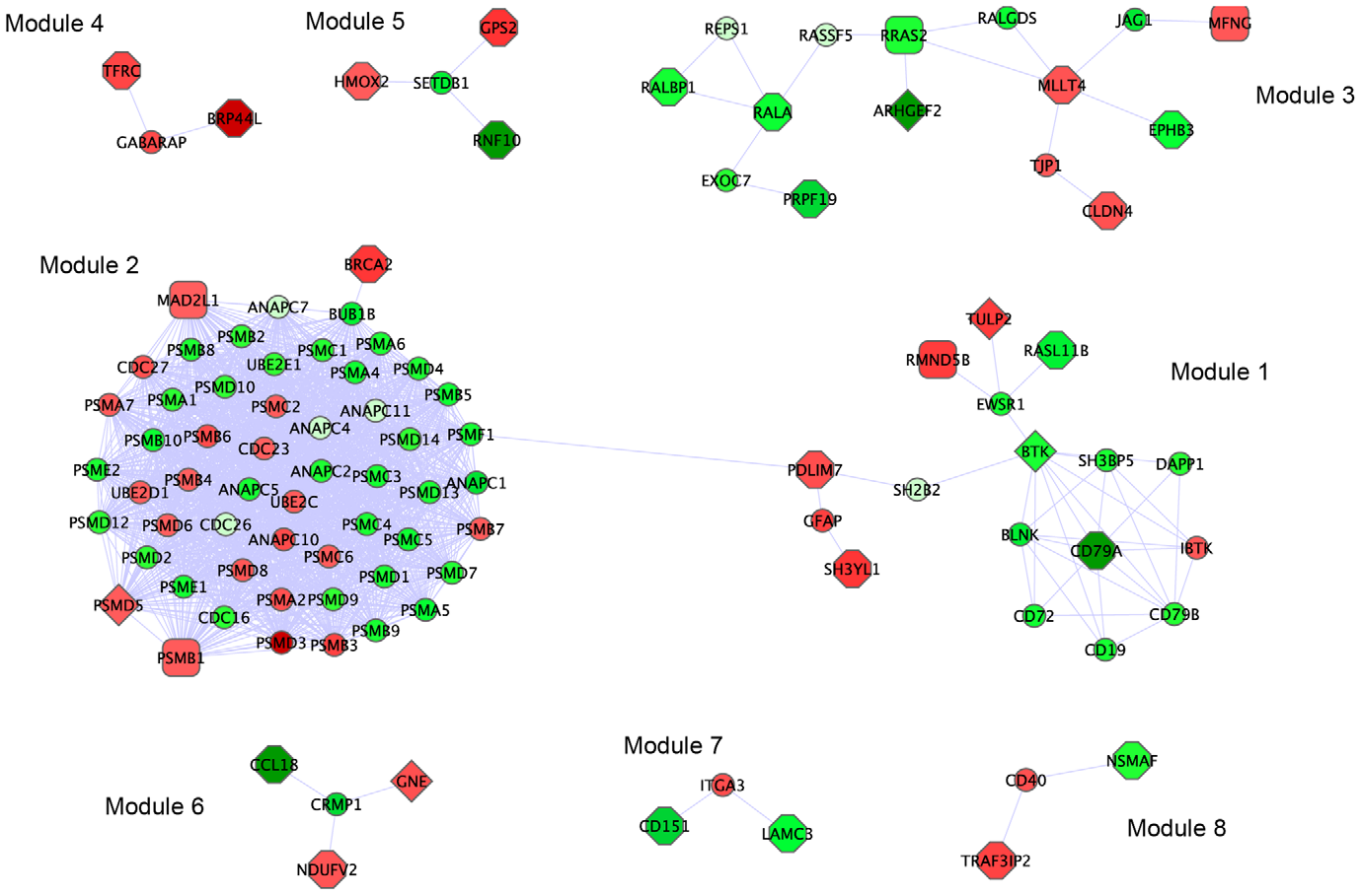
Netbox modules identified using the integrated PFS signature. Different modules are spatially separated for visualization. The genes present in our signature are shaped as octagons (mRNA features), diamonds (methylation features) and rectangles (copy number feature). The linker nodes are represented as small circles. Nodes are colored based on the mRNA expression profile of different genes (green: down-regulated in short recurrence patients (PFS<6mo) compared to long recurrence (PFS>40mo), and red: up-regulated).

Secondly, Netbox (Figure 7) identified eight modules of connected genes, ranging in size from three to 54 genes. Some of the interactions identified by Netbox were common with those identified by IPA (e.g., the module containing RALA, RRAS2, RALBP1, ARHGEF2). To annotate the biological function of these modules, we assessed over-representation of genes in each module using IPA (Table S2). Module 1 was enriched in genes involved in PI3K signaling in B lymphocytes, FcγRIIB signaling in B lymphocytes and p70S6K/mTOR signaling; module 2 was enriched in genes involved in the protein ubiquitination pathway, protein kinase A signaling, and DNA double-strand repair by homologous recombination; and, module 3 was enriched among others, for genes involved in phosphoslipase C signaling, pancreatic adenocarcinoma signaling, and tight-junction signaling.

The genes below have been reported as implicated in cancer and were identified by one or both of the pathway analysis tools.

**PGRMC1** (Progesterone Receptor Membrane Component 1; Xq24; mRNA) was down-regulated in patients with short recurrence time. Interestingly, depleting PGRMC1 in ovarian tumors makes these tumors more resistant to cisplatin treatment (consistent with our observation) [18], and this gene is down-regulated in breast cancer specimens compared to control tissues [19]. **CITED2** (Cbp/p300-interacting transactivator with Glu/Asp-rich carboxy-terminal domain 2; 6q24.1; mRNA) up-regulation is associated with shorter recurrence times. Knockdown of CITED2 in cell lines results in increased sensitivity to cisplatin, which makes it a candidate for targeted therapy for SeOvCa [20]. **TFRC** (Transferrin receptor; 3q29; mRNA) is up-regulated in patients with shorter recurrence times, which is consistent with its behavior in esophageal squamous cell carcinoma, where TFRC up-regulation is associated with worse prognosis [21]. **RALBP1** (ralA binding protein 1; 18p11.22; mRNA) acts as a transporter of glutathione conjugates and chemotherapeutic drugs and serves as a link between G-protein and tyrosine kinase signaling and drug resistance [22]. In SeOvCa, **RALA** (ral-A; 7p14.1; mRNA) and RALBP1 are down-regulated in patients with short recurrence time compared to long-recurrence time. **ALOX12** (arachidonate 12-lipoxygenase; 17p13.1; METH) acts as a methylation marker (hypermethylation) in pancreatic cancer genome [23] and hypermethylation of ALOX12 is predictive of overall survival (poor prognosis) in patients with acute myeloid leukemia [24]. In the TCGA SeOvCa data set, ALOX12 is hypermethylated in patients with longer recurrence intervals. **ARHGEF2** (rho/rac guanine nucleotide exchange factor 2; 1q22; METH) is a member of the Dbl family of Rho activators and it has Rho-specific GDP/GTP exchange activity for RhoA [25]. Activated RhoA contributes to cancer progression by transducing various signals into downstream signaling cascades, such as cytoskeleton reorganization, cellular invasion, and cell proliferation [25]. Increased ARHGEF2 expression contributes to the tumor progression phenotype associated with p53 mutation [26]. **ID4** (inhibitor of DNA binding 4; 6p22.3; METH) belongs to the ID family of transcription factors, and its methylation status acts as a prognostic biomarker in some cancers [27]. In TCGA data, lower methylation beta values and higher expression are associated with shorter recurrence times. ID4 has been identified as a transcriptional target of the protein complex mutant p53/E2F1/p300 in breast cancer [28]. Some of these genes are candidates for targeted experiments.

#### C. Potential Biomarkers and Therapeutic Targets for Ovarian Cancer

Identifying biomarkers and therapeutic targets for SeOvCa patients is a challenge given the complexity and heterogeneity of genomic alterations in this cancer. In order to suggest possible biomarkers and therapeutic targets, we ranked the 156 features in the PFS signature based on their individual power (Table S3). Based on the probability distribution of each of features, we stratified all SeOvCa patients into three categories: low (bottom 15% values), intermediate, and high (top 15% values).

**Figure 8:**
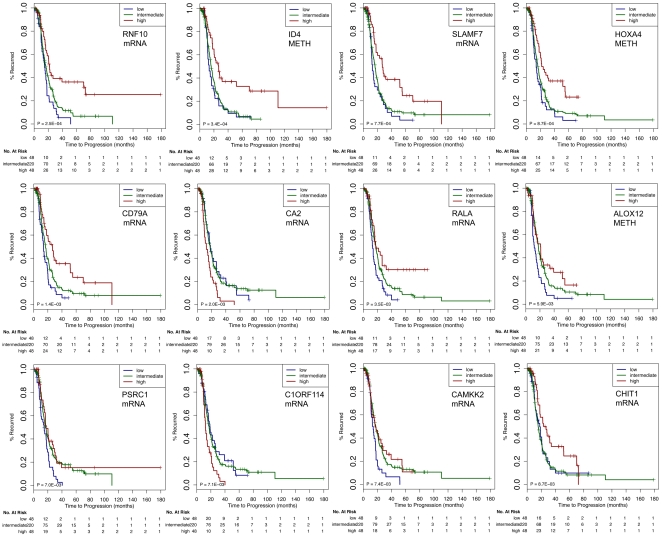
Features from the integrated PFS signature ranked based on their stratification performance. Top ranked features (categorized based on their values from the respective data type as low [bottom 15%], intermediate and high [top 15%]) could potentially act as biomarkers and therapeutic targets.

Out of the 12 most discriminant features in the integrated PFS signature, ID4, CA2, and C1ORF114 are up-regulated in tumors with short recurrence times, and RNF10, SLAMF7, HOXA4, CD79A, RALA, ALOX12, PSRC1, CAMKK2, and CHIT1 are down-regulated in tumors with short recurrence times (Figure 8). In addition to ID4 and ALOX12, which are discussed above, several of these genes are known to be implicated in cancer:

**RNF10** (ring finger protein 10; 12q24.31) has been implicated in cellular processes such as signal transduction, transcriptional regulation, ubiquitination and apoptosis [29], [30]. RNF10 expression (mRNA) is correlated with DNA copy-number, and in a global analysis with all microRNAs, we found that RNF10 expression was most strongly anti-correlated with miR-92A (cc = −0.14). RNF10 is a predicted target of miR-92a, (Targetscan pct score = 0.75). **SLAMF7** (SLAM family member 7; 1q23.3) down-regulation is associated with decreased phosphorylation of ERK1/2, STAT3 and AKT, as well as altered phosphorylation of multiple kinases, inducing signaling cascade in multiple myeloma [31]. In TCGA data, SLAMF7 is down-regulated in patients with faster recurrence and its expression is not clearly associated with either CNA data or DNA methylation. TCGA data is suggestive of possible targeting by miR-129-5p (cc = −0.18; pct = 0.33). **HOXA4** (homeobox A4; 7p15.2) is highly expressed in invasive ovarian carcinomas compared to benign or borderline (non-invasive) carcinoma [32]. In acute myeloid leukemia, low expression of HOXA4 is a favorable outcome predictor [33]. In TCGA data HOXA4 is methylated, and down-regulation is associated with samples that show faster tumor recurrence. **CAMKK2** (calcium/calmodulin dependent protein kinase kinase 2; 12q24.31) is down-regulated in tumors with short-recurrence time in TCGA data. Activating CAMKK2 in cervical cancer inhibits human cancer cell growth in both LKB1-expressing and LKB1-deficient cervical cancer cells [34]. This suggests that CAMKK2 activation could indicate improved prognosis of ovarian cancer patients. We suggest that these genes are reasonable candidates for biomarker studies in SeOvCa.

## Conclusions

We have made substantial progress in outcome prediction by using data integration, rather than just a single genomic data type, and by analyzing as many as 500 cases, more than the ∼150 or so available to earlier studies [3], [5]. In part, the advance was made possible by the Cancer Genome Atlas (TCGA) project, which profiled more than 500 primary surgical samples from serous ovarian carcinoma patients for copy number, microRNA and mRNA expression, and DNA methylation, and provided clinical information about disease recurrence and survival. We used this dataset to perform both discrete stratification analysis and continuous clinical time predictions.

To avoid over-fitting and to facilitate interpretation, we reduced 50,000 molecular features to fewer than 200, which are most associated with tumor recurrence and patient survival. The integrated PFS signature provided better prediction than signatures based on individual data types. The predictive performance of the integrated PFS signature was independent of the method of stratification into discrete risk categories.

Our results demonstrate that signatures based on multiple data types can be more powerfully predictive than those based on a single data type and this may be true for other tumor types as well. For serous ovarian cancer, we provide a new prediction tool for patient-specific time to recurrence and survival that can be used by physicians to predict likely disease progression following surgery and molecular profiling. In addition, the gene signatures identified and pathways differentially affected in patients more resistant to standard therapy, may prove useful for the discovery of therapeutic targets in the context of efforts to improve therapy for high-grade SeOvCa patients. In particular, our feature ranking method identified RNF10, ID4, SLAMF7, HOXA4, ALOX12 and CAMKK2 (among others) as the potentially most interesting biomarkers and therapeutic targets.

## Materials and Methods

Traditionally, a univariate Cox proportional hazards regression model is used to relate expression to outcome. In this method, significant genes are selected based on arbitrary p-value cut-offs and thresholding of the associated Wald z-statistic. A training cohort is used to compute risk scores followed by strata creation based on thresholding of these scores. The limitations of this approach include not just the arbitrariness of the imposed stratification, but also the arbitrarily chosen p-value cut-offs.

An alternative approach is to use penalized proportional hazards (PH) regression, including the L_1_ (Lasso) and L_2_ penalized estimation (Ridge regression). Including all genes in the predictive model introduces noise and can lead to a poor predictive model. The L_1_-based PH regression performs feature selection and shrinkage simultaneously, and appears to outperform the univariate Cox approach [35], [36]. Here, we implemented an L_1_-regularized Cox proportional hazards model to do feature selection using the Cox model with an L_1_ penalty, as proposed by Park and Hastie [11].

Given the availability of clinical times, in addition to predicting discrete patient risk stratification, we implemented an algorithm to directly compute the continuous variables, the clinical *times-to-event* (PFS and OS) based on an algorithm discussed in Heller and Simonoff [37]. In an earlier study [38], an accelerated failure time model was used to predict median survival times for patients with progressive metastatic disease using clinicopathological factors. The estimated concordance index for the validation data was reported to be 0.67, with substantial variability in the actual survival among patients with similar predicted median times. In another study [39], a nomogram based on a Cox model was constructed for finding patient-specific probabilities of metastasis-free survival in patients with recurrent prostrate cancer following surgery and/or radiation therapy resulting in prostate-specific antigen level as a prognostic marker. A bootstrap concordance index was computed to assess the performance of the prognostic marker and was reported to be 0.69.

All data in TCGA including the data used in this study [1] have the appropriate IRB consent. Details of the methods used in this study are as follows:

### Multivariate Cox Regression model

The CoxPath model [11] is a path following algorithm for the L_1_-regularized Cox proportional hazards model. Here, the coefficients (β) for the predictors (x′) are estimated by solving a set of non-linear equations that satisfy the maximum likelihood criterion
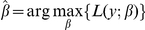
The partial likelihood function (L) with respect to the given data *{(x_i_, y_i_):i = 1, …., n}* is:
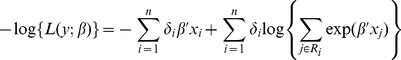
where R_i_ is the risk set at time y_i_ and δ_i_ is a binary variable for censored data. Analogous to Lasso [40], which adds a complexity penalty term to the squared error loss criterion, the CoxPath is modified with regularization as:
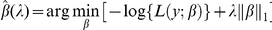
where *λ>0* is the regularization parameter.

A predictor-corrector algorithm is used to determine the entire path of the coefficient estimates as λ varies, i.e., find β(*λ*) starting from *λ = λ_max_* to *λ = 0*; where *λ_max_* is the largest *λ* that makes β(*λ*) non-zero. The algorithm computes a series of solution sets, each time estimating the coefficients with a smaller *λ* based on the previous estimate. The solution paths are calculated that satisfy
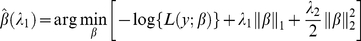
where *λ_1_ ε (0, ∞)*. *λ_2_* is a fixed, small, positive constant as referred to in the original Park and Hastie reference [11] which removes degeneracy and instability due to strong correlations between covariates. When the correlations are not strong, the effect of the quadratic penalty with a small *λ_2_* is negligible.

We employ the concordance probability estimate (CPE) for parameter tuning in the CoxPath model. Two-fold cross-validation, repeated 10 times, is performed to obtain concordance probability estimates (CPE) at different values of the regularization parameter. The optimal parameter is chosen that maximizes the CPE in the cross-validation procedure.

### The concordance probability estimate (CPE)

The predictive strength of the CoxPath algorithm was assessed using the concordance probability estimate (CPE). Gönen and Heller [41] derived an analytic expression for the concordance probability in the Cox proportional hazards model calculated as:
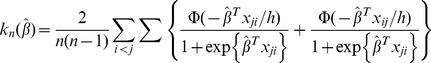
where *x_ij_* represents the pairwise difference *x_i_* - *x_j_*; *h* is a scaling parameter that is used to smooth the CPE and Φ is a local distribution function. The concordance probability is used to evaluate the discriminatory power and the predictive accuracy of the Cox proportional hazards model. A concordance probability of 1.0 represents a model that has perfect discrimination, and a value of 0.5 indicates a random prediction. A strong concordance signifies that the baseline factors in the Cox model are highly informative in understanding the relative risk of disease-recurrence between any two patients at time *t*.

We used two methods for evaluating the predictive performance of the derived prognostic signatures. One approach predicts the patient-risk stratification utilizing the features estimated by Lasso-Cox approach (along with the calculation of hazard ratios and p-values). A second approach predicts the clinical *time-to-event* using an algorithm discussed by Heller and Simonoff [37].

### The Prognostic Scores

#### c-score

A prognostic index based on the linear predictor (cox-score) for each patient is calculated as:
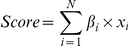
where x_i_ is the value of each gene (or microRNA) in the signature and β_i_ is the estimated regression coefficient of that gene obtained from the CoxPath model. Patients were stratified into three groups (*low*-, *intermediate*- and *high*-risk groups) using tertile stratification based on the training data. The c-scores and the cut-offs derived from the training cohort were applied directly to test cohorts and were not re-estimated.

#### t-score

The prognostic index based on t-score was calculated as the difference in the average of the poor prognosis genes with the average of the good prognosis genes for each tumor profile based on the genes obtained from the CoxPath model [11].

### Time to Recurrence Prediction

Fitting a regularized multivariate regression model using Lasso-Cox allows us to compute the predicted median *time-to-event*. For this purpose, we utilized the median failure time model developed by Heller and coworkers [37]. The median *time-to-event* for a given X is where
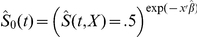
For a given covariate profile, the predicted median time refers to the time point that one would predict 50% of the cohort to survive beyond.

### Data Integration

Genomic data integration, the process of statistically combining diverse sources of information from multiple data types to make large-scale predictions, is becoming increasingly prevalent. In performing integration, it is advisable to assess the degree to which predictive power increases with the addition of more features and to investigate the biological interpretation of the resulting features. Various integration methods are available that include kernel space integration [42] for machine learning analysis, sparse canonical correlation analysis [43], and iCluster [44]. We are interested in a method that works with as few features as possible, is amenable to biological interpretation of the resulting discriminant features and optimizes association with outcome measures.

There are various mechanisms that regulate gene expression. These include DNA methylation, histone modification, DNA copy-number gain/loss, and targeting by transcription factors and microRNAs. Using features derived from integration of various data types may lead to richer biologically relevant information than the analysis of a single data type.

In summary, to overcome possible limitations of other integration methods, we have created an integration method that reduces the dimensionality of the feature space with the intent that the resulting features are biologically significant. The vector space integration used here (Figure 2 and Supplementary File S5) is similar to integration approaches in support vector machine analysis [45]. We began by computing Spearman rank correlations among different data types: mRNA and copy-number; mRNA and DNA methylation; and mRNA and microRNA, respectively, as these data types are not completely independent of each other. Next we used three separate cut-offs respectively for each potentially correlated data pair to filter the features for input into Cox Lasso cross-validation analysis. Our aim was to identify potential targets for therapy in the context of multiple genomic characteristics and to provide more accurate prognostic and predictive assessment than is possible without data integration.

## Supporting Information

Table S1 Biological function and disease association analysis for the integrated PFS signature using IPA.
(TXT)

Table S2 Over-representation analysis of modules identified by the Netbox algorithm for the integrated PFS signature using IPA.
(TXT)

Table S3 Ranked features for the PFS signature.
(TXT)

File S1 Supporting information on mRNA expression data for “Time to recurrence and survival in serous ovarian tumors predicted from integrated genomic profiles”.
(PDF)

File S2 Supporting information on DNA methylation data for “Time to recurrence and survival in serous ovarian tumors predicted from integrated genomic profiles”.
(PDF)

File S3 Supporting information on microRNA expression data for “Time to recurrence and survival in serous ovarian tumors predicted from integrated genomic profiles”.
(PDF)

File S4 Supporting information on copy number alteration data for “Time to recurrence and survival in serous ovarian tumors predicted from integrated genomic profiles”.
(PDF)

File S5 Supporting information on integrated data for “Time to recurrence and survival in serous ovarian tumors predicted from integrated genomic profiles”.
(PDF)
