# Supplementary material for: Time to Recurrence and Survival in Serous Ovarian Tumors Predicted from Integrated Genomic Profiles
Source: PLoS One. 2011 Nov 3;6(11):e24709. doi: 10.1371/journal.pone.0024709 (PMC3207809; doi:10.1371/journal.pone.0024709)
Supplement: File S1 — Supporting information on mRNA expression data for “Time to recurrence and survival in serous ovarian tumors predicted from integrated genomic profiles”. (PDF) [file pone.0024709.s004.pdf]

Time to Recurrence and Survival in Serous Ovarian Tumors Predicted from Integrated Genomic Profiles

Parminder K. Mankoo, Ronglai Shen, Nikolaus Schultz, Douglas A. Levine and Chris Sander

mRNA Gene Expression

Data from three different microarray expression platforms (Agilent 244K, Affymetrix Exon and Affymetrix U133A) were combined and normalized as reported in the TCGA paper [1]. All 11,864 genes from the unified TCGA dataset (derived from genes represented on all three platforms) were utilized for Cox Lasso regression analysis. All mRNA expression datasets were mean-centered. Mean-centered expression values were calculated by subtracting the mean expression value across patients from the gene estimate and dividing by the standard deviation across patients.

External data preparation Four external mRNA expression datasets were used to evaluate the performance of TCGA-data based signatures:

Tothill data: mRNA expression data for 243 tumor samples and 17255 genes [3]. We used only the 174 high-grade serous ovarian cancer samples to match the TCGA cohort.
Dressman data: mRNA expression data with overall survival information for 117 tumor samples and 12113 genes [4].
Bonome data: mRNA expression data for 166 tumor samples and 12114 genes [5]. Overall survival data was available for 163 patients and progression free survival data for 138 patients (clinical data as of February 4, 2008).
Yoshihara & Tanaka data: mRNA expression data for 110 patients and 18177 probes [6]. For each gene with multiple probes, we selected the probe with the largest sum of the squares of the individual expression values, resulting in 13154 unique genes.

Progression Free Survival: At λ=.830, we selected 181 features (Table ST4 [7]), cv.CPE = 0.79. Tertile stratification on the training data led to a p-value of 0.17 (c-score) and t-score stratification led to a p-value of 0.05, CPE.test = 0.77. Using the Tothill data set, tertile stratification based on c-score led to a p-value = 0.035 (and t-score p-value = 0.012) and CPE.tothill = 0.77. Our gene signature was not applicable to the Yoshihara & Tanaka dataset, as gene expression values were only available for 112 of the 181 genes in the progression-free-survival signature (Figure 1SA, 1SB).
Survival: At λ=.839, we selected 219 features (Table ST5 [8]), cv.CPE= 0.81. Tertile stratification based on c-score led to a p-value = 0.70 and t-score stratification led to a p-value of 0.09, CPE.test = 0.80. Using the Bonome dataset, tertile stratification led to a p-value = 0.05 (c-score) and p=0.18 (t-score) and CPE.bonome=0.75; using the Tothill dataset, tertile stratification led to a p-value = 0.048 (c-score) and p=0.014 (t-score), and CPE.tothill= 0.78; and using the Dressman dataset, tertile stratification led to a p-value = 0.008 (c-score) and p=0.033 (t-score). Our survival signature was not applicable to

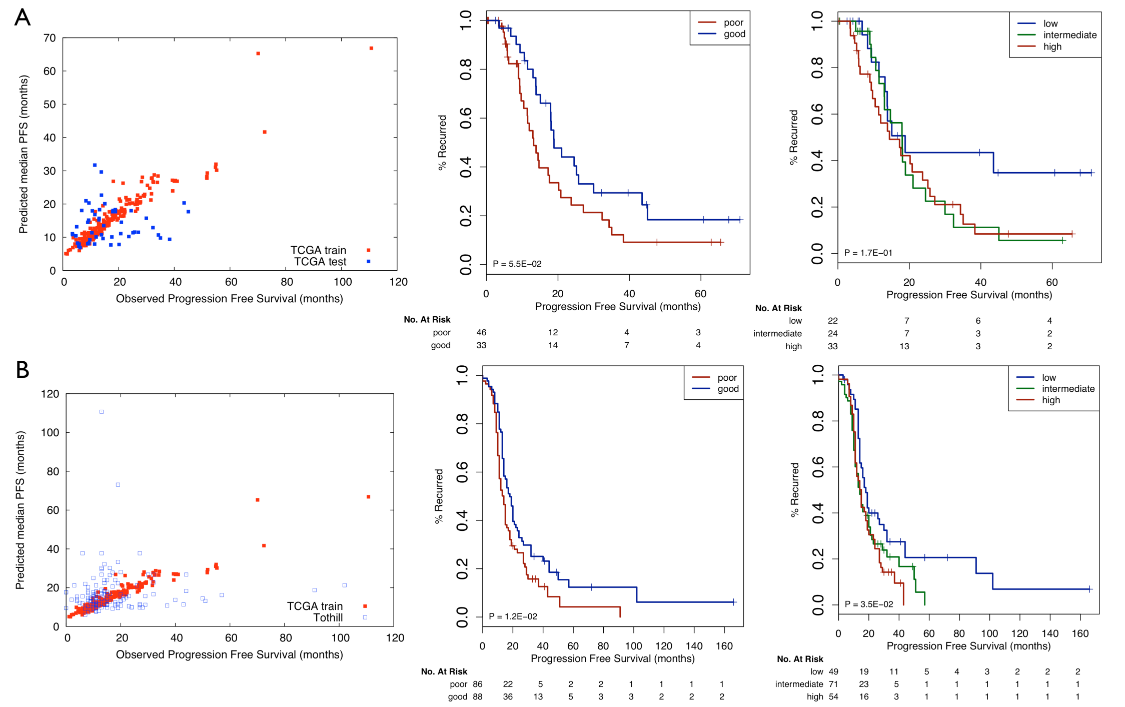

Figure 1SA: Time Prediction and Kaplan-Meier plots from PFS mRNA data: Median *time-to-event* prediction for uncensored data and KM plots of test data using t-score stratification (left), and c-score stratification (right) for: (A). TCGA test data; and (B) Tothill data.

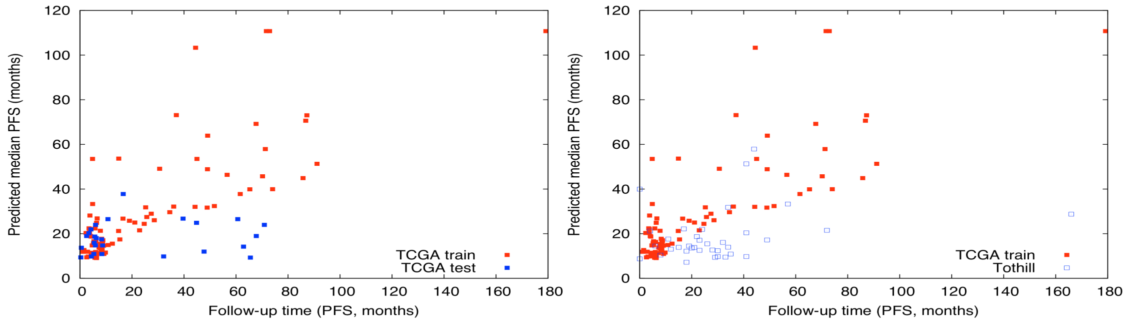

Figure 1SB: Time prediction for censored data from PFS mRNA signature: TCGA test data (left) and Tothill mRNA data (right).

Yoshihara & Tanaka dataset as gene expression values were only available for 143 of the 219 genes in the overall survival signature (Figures 1SC, 1SD).

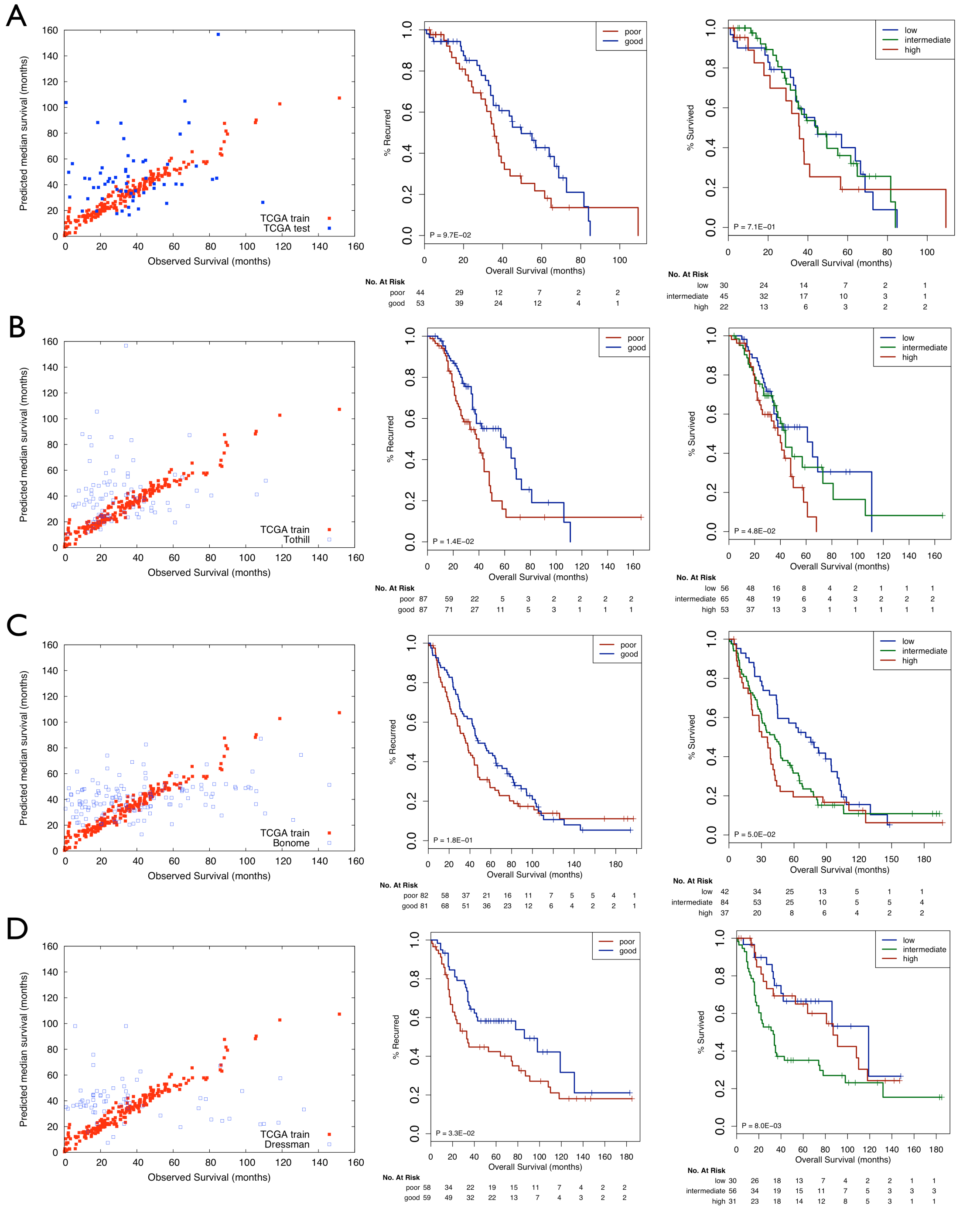

Figure 1SC: Time Prediction and Kaplan-Meier plots from OS mRNA signature: (A) TCGA test data; (B) Tothill dataset; (C) Bonome dataset and (D) Dressman dataset. KM plots using t-score (left) and c-score (right).

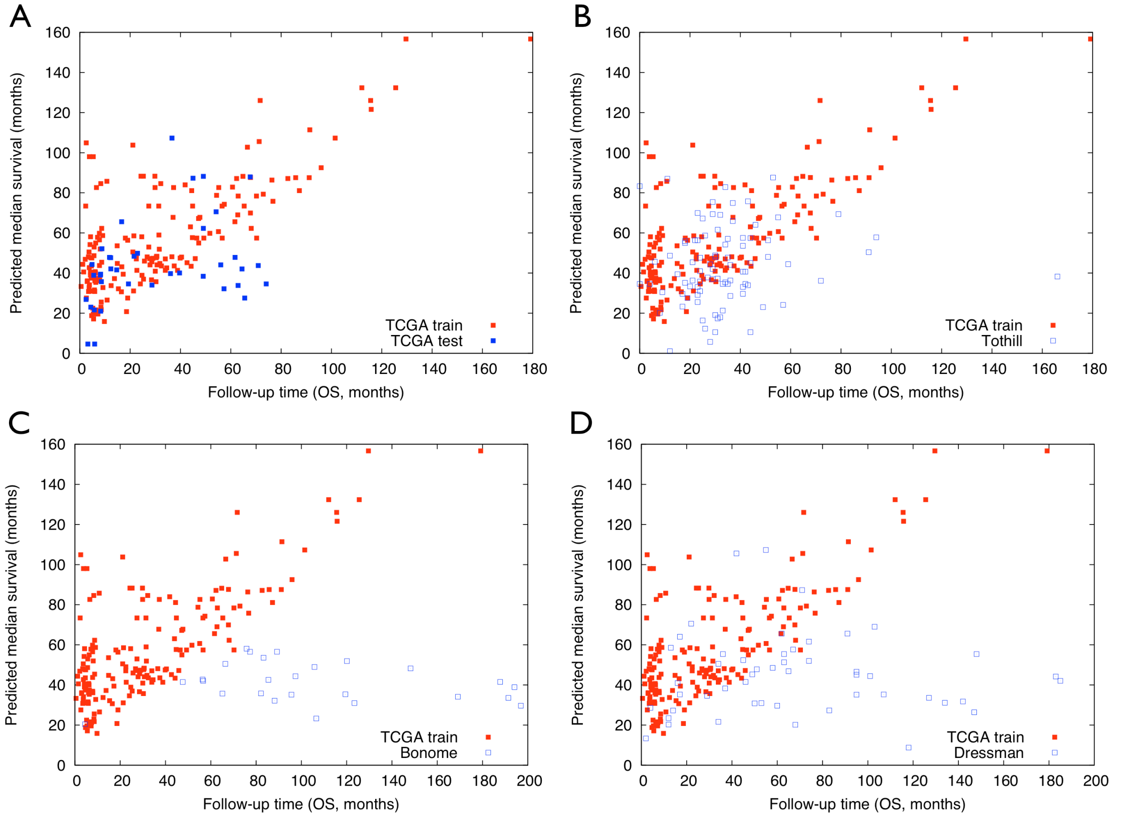

Figure 1SD: Prediction of median time-to-event for censored data from mRNA expression signatures: Median *time-to-event* prediction and comparison with follow-up times: A). TCGA test data; (B) Tothill dataset; (C) Bonome dataset; and, (D) Dressman dataset.

Functional and Disease association for genes in mRNA signatures

In order to prioritize functionally relevant genes and statistically and significantly associated canonical pathways within our mRNA signatures, we utilized Ingenuity pathway analysis (IPA) [7].

mRNA PFS signature: The top significant canonical pathways from mRNA TTP signature include: Granzyme A signaling; cardiomyocyte differentiation via BMP receptors; glycerolipid metabolism; Angrin interactions at neuromuscular junction; B cell development; HER-2 signaling in breast cancer and such (Figure 1SE). Other important categories (not statistically significant) were Androgen signaling and DNA double-strand break repair by homologous recombination. Statistically significant biological function categories include cell-to-cell signaling and interaction; molecular transport; lipid metabolism; cell death; cancer; and DNA replication, recombination and repair.

mRNA OS signature:

The top significant canonical pathways from the mRNA OS signature include: LXR/RXR activation; IL-6 and IL-10 signaling; PKA and p38MAPK signaling; TNFR1 signaling and PPAR signaling and such (Figure 1SF). General biological function categories (statistically significant) include: Inflammatory disease; lipid metabolism; drug metabolism, reproductive system disease; cell-to-cell signaling and interaction; and DNA replication, recombination and repair etc.

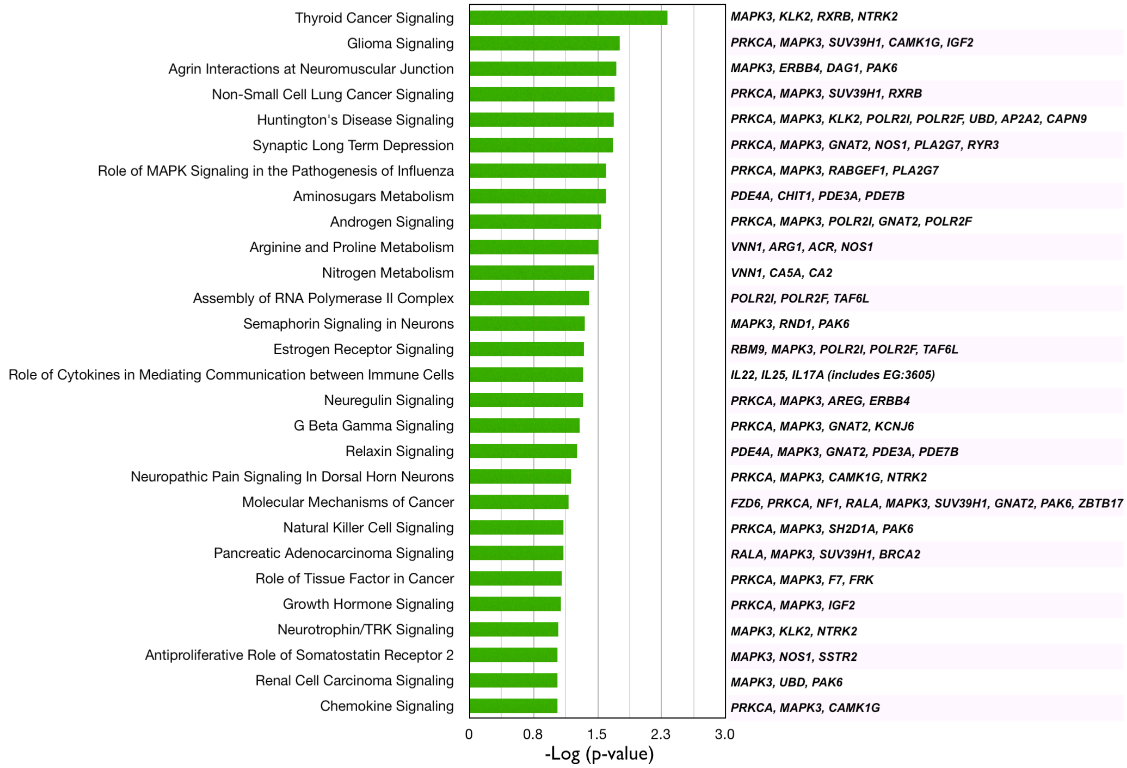

Figure 1SE: Canonical pathway analysis of 181 PFS mRNA features: Top functions with p-value cut-off < 0.1 are depicted.

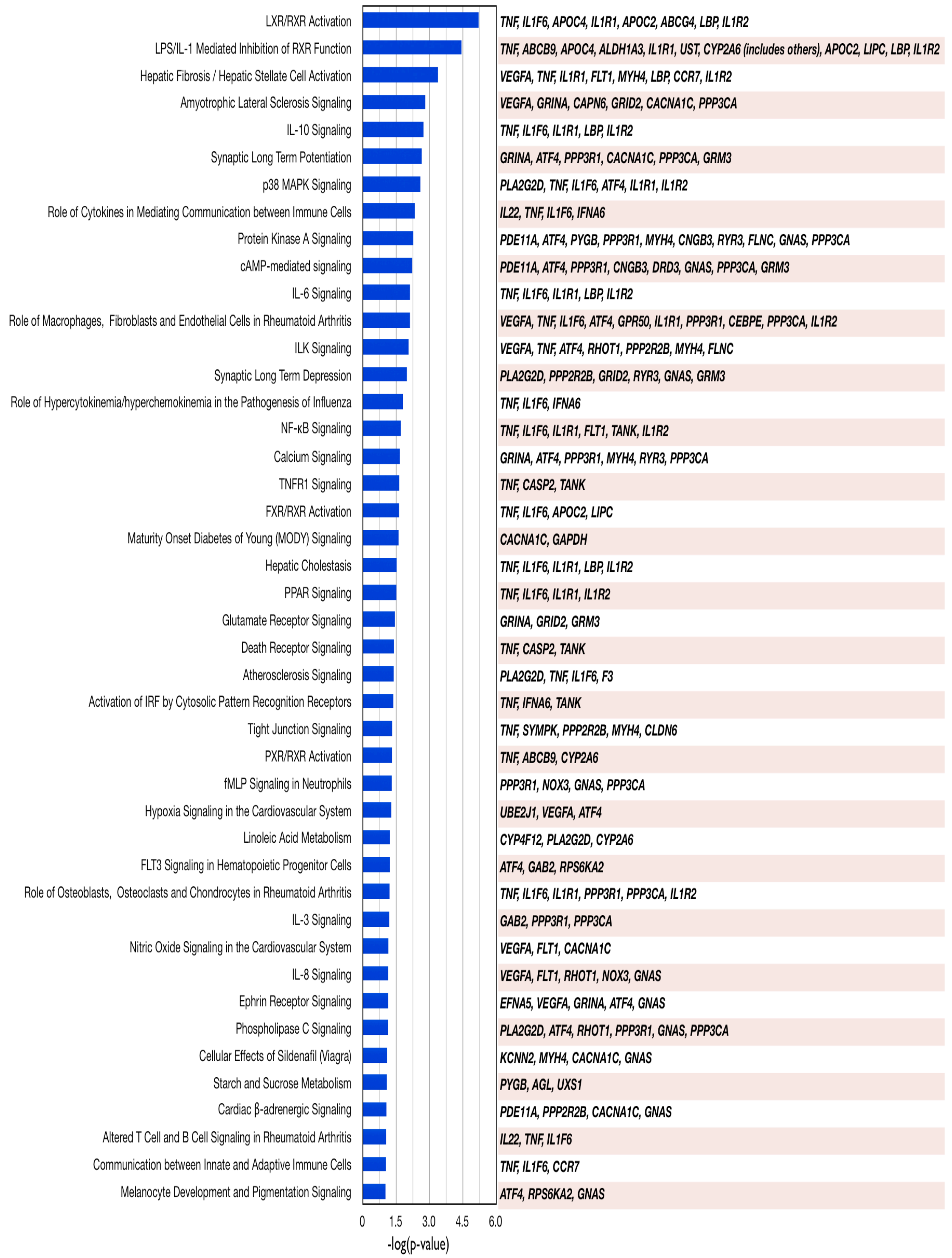

Figure 1SF: Canonical pathway analysis of 219 OS mRNA features: Top functions with p-value cut-off < 0.1 are depicted.

References:

1. TCGA (2011) Integrated Genomic Analyses of Ovarian Carcinoma. Nature 474: 609-615.
2. Park MY, Hastie T (2007) L1-regularization path algorithm for generalized linear models. J R Statist Soc B 69: 659-677.
3. Tothill RW, Tinker AV, George J, Brown R, Fox SB, et al. (2008) Novel molecular subtypes of serous and endometrioid ovarian cancer linked to clinical outcome. Clin Cancer Res 14: 5198-5208.
4. Dressman HK, Berchuck A, Chan G, Zhai J, Bild A, et al. (2007) An integrated genomic-based approach to individualized treatment of patients with advanced-stage ovarian cancer. J Clin Oncol 25: 517-525.
5. Bonome T, Levine DA, Shih J, Randonovich M, Pise-Masison CA, et al. (2008) A gene signature predicting for survival in suboptimally debulked patients with ovarian cancer. Cancer Res 68: 5478-5486.
6. Yoshihara K, Tajima A, Yahata T, Kodama S, Fujiwara H, et al. Gene expression profile for predicting survival in advanced-stage serous ovarian cancer across two independent datasets. PLoS One 5: e9615.
7. http://cbio.mskcc.org/~mankoo/ST4.txt
8. http://cbio.mskcc.org/~mankoo/ST5.txt
9. IPA Ingenuity Pathway Analysis; http://www.ingenuity.com.
