# Supplementary material for: Time to Recurrence and Survival in Serous Ovarian Tumors Predicted from Integrated Genomic Profiles
Source: PLoS One. 2011 Nov 3;6(11):e24709. doi: 10.1371/journal.pone.0024709 (PMC3207809; doi:10.1371/journal.pone.0024709)
Supplement: File S2 — Supporting information on DNA methylation data for “Time to recurrence and survival in serous ovarian tumors predicted from integrated genomic profiles”. (PDF) [file pone.0024709.s005.pdf]

Time to Recurrence and Survival in Serous Ovarian Tumors Predicted from Integrated Genomic Profiles

Parminder K. Mankoo, Ronglai Shen, Nikolaus Schultz, Douglas A. Levine and Chris Sander

DNA methylation

DNA methylation is an important mechanism in the regulation of gene function and serves as an epigenetic marker for tumor detection, classification, and prognostication. DNA hypermethylation can occur in the cytosines that precede a guanosine (referred to as CpG dinucleotides) to form methyl cytosine (5-methylcytosine). CpG dinucleotides are found at increased frequency in the promoter region of many genes [1]. The Illumina Infinium DNA methylation assay was performed on 519 TCGA ovarian samples and eight fallopian tube samples as part of TCGA study [2]. The beta value DNA methylation scores are calculated as (M/(M+U)), where M is the intensity of methylated and U is the intensity of unmethylated bead type for each CpG locus. We used the level 3 DNA methylation data available for 7624 genes. Next, we computed Spearman rank correlations of these genes with mRNA expression data for the training set comprising of 316 and 384 samples, respectively, associated with each outcome measure, PFS and OS,

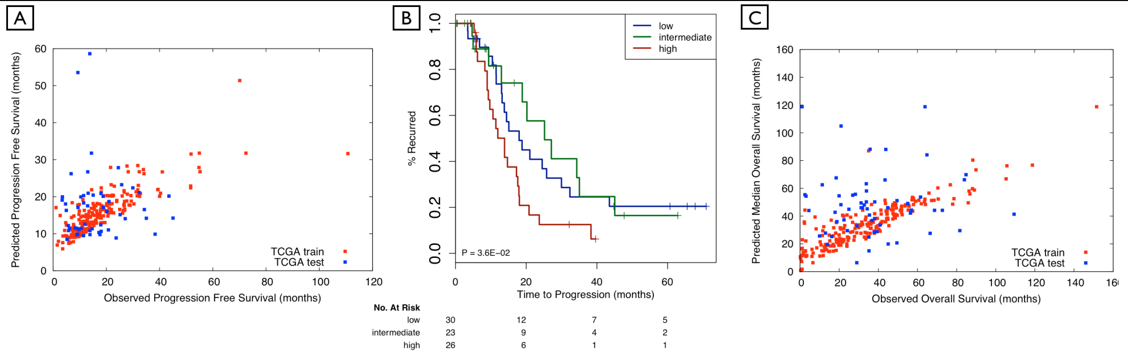

Figure 2SA: Performance of PFS and OS DNA methylation signatures: (A) Median *time-to-event* prediction for uncensored data from PFS DNA-methylation signature; (B) Kaplan-Meier plot from PFS signature; and (C) median *time-to-event* prediction for OS DNA-methylation signature.

respectively. A cut-off of -0.05 led to 4564 and 4642 genes that were selected for further analysis of PFS and OS, respectively.

<u>Progression Free Survival</u>: At λ=.838, we selected 140 features (Table ST6 [3]). Tertile stratification led to a p-value = 0.03 (c-score), CPE.test = 0.72 (Figures 2SA, 2SB).
<u>Survival</u>: At λ=.858, we selected 171 features (Table ST7 [4]). Tertile stratification led to a p-value = 0.52 (c-score), CPE.test = 0.74 (Figures 2SA, 2SB).

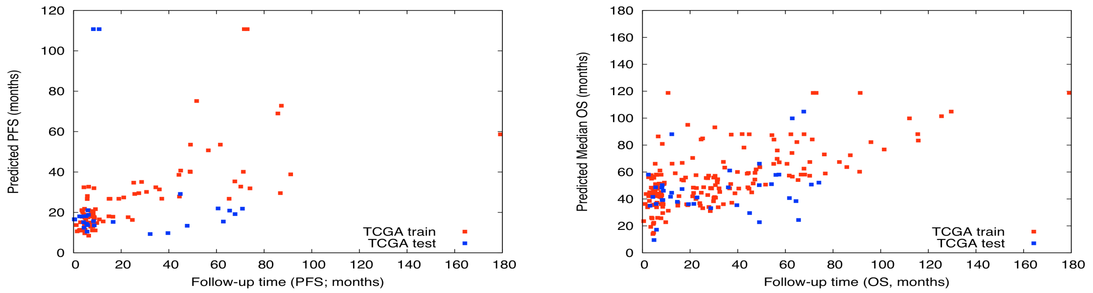

Figure 2SB: Performance of PFS and OS DNA methylation signatures for censored data: Time prediction from PFS signature (left) and time prediction from OS signature (right).

Functional and Disease association for genes in DNA methylation signatures

<u>*Progression Free Survival*</u>: The top statistically significant canonical pathways include FXR/RXR and LXR/RXR activation; G Beta Gamma Signaling; Hepatic Cholestasis; Tight Junction Signaling; Cell Cycle: G2/M DNA damage and checkpoint regulation; primary immunodeficiency signaling; PPAR and TREM1 signaling; IL12 and IL10 signaling; hereditary breast cancer signaling; Glucocorticoid receptor signaling; and Notch signaling (Figure 2SC). The top and statistically significant toxicity pathways

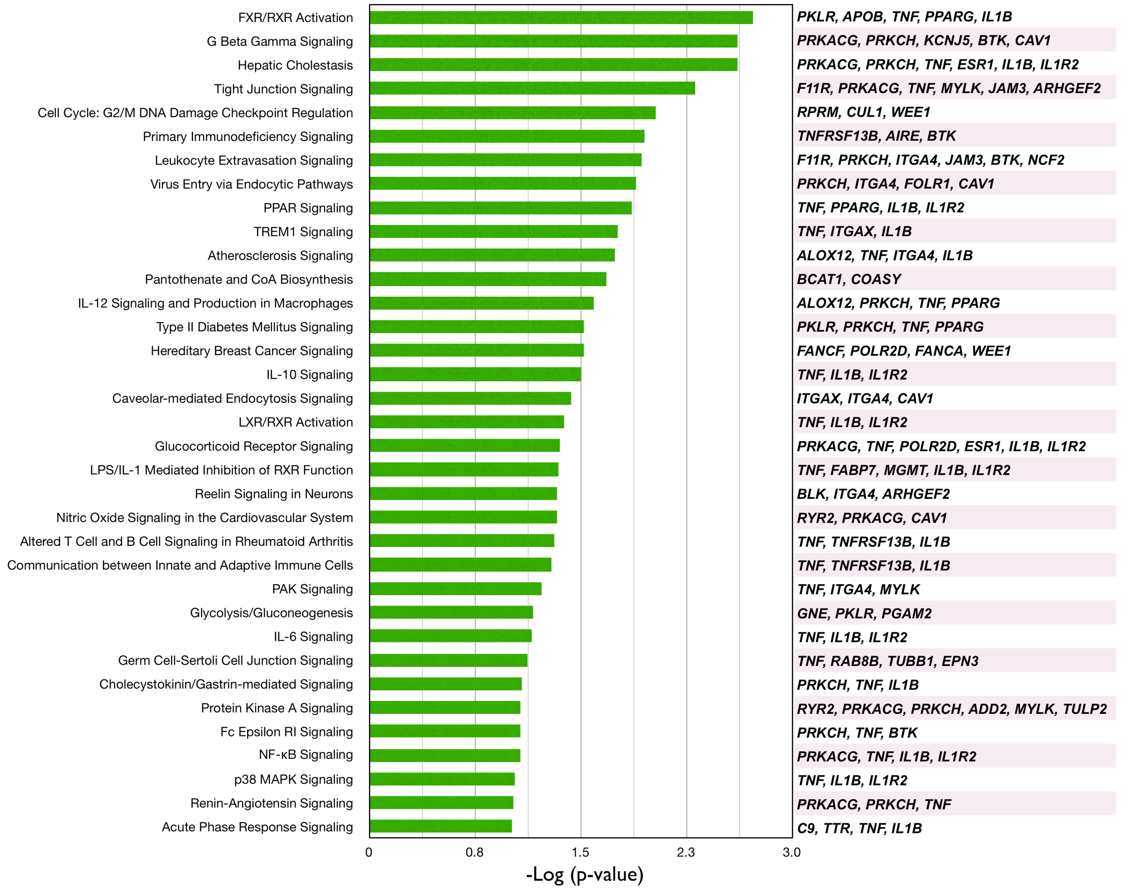

Figure 2SC: Canonical pathway analysis of 140 PFS DNA methylation features: Top functions with p-value cut-off < 0.1 are depicted.

include mechanism of gene regulation by peroxisome proliferators via PPARα; increased renal proliferation; FXR/RXR activation, cell cycle: G2/M DNA damage checkpoint regulation, NFKB signaling.

Overall Survival: The top statistically significant canonical pathways include: eicosanoid signaling; intrinsic prothrombin activation pathway; tryptophan metabolism; GABA receptor signaling and β-alanine metabolism (Figure 2SD). Statistically significant general biological functions include: lipid metabolism; genetic disorder; amino acid and drug metabolism; cellular growth and proliferation; cancer; DNA recombination, replication and repair; cell cycle; RNA damage and repair, etc.

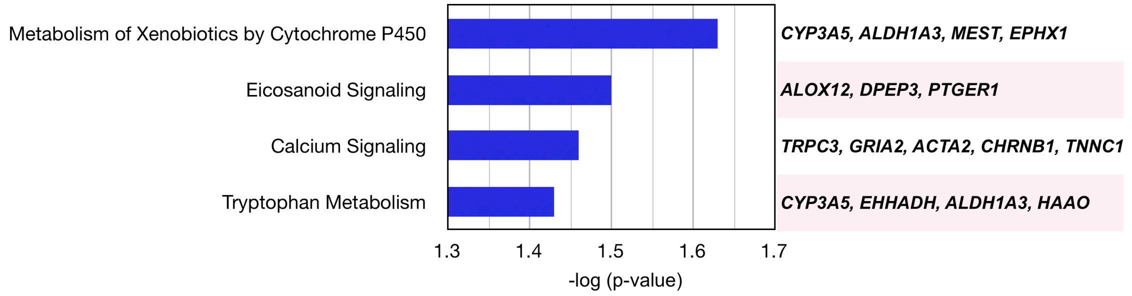

Figure 2SD: Canonical pathway analysis of 171 OS DNA methylation features: Top functions with p-value cut-off < 0.1 are depicted.

References:

1. Shih Ie M, Chen L, Wang CC, Gu J, Davidson B, et al. Distinct DNA methylation profiles in ovarian serous neoplasms and their implications in ovarian carcinogenesis. Am J Obstet Gynecol 203: 584 e581-584 e522.

2. TCGA (2011) Integrated Genomic Analyses of Ovarian Carcinoma. Nature 474: 609-615.

3. http://cbio.mskcc.org/~mankoo/ST6.txt

4. http://cbio.mskcc.org/~mankoo/ST7.txt
