# Supplementary material for: Time to Recurrence and Survival in Serous Ovarian Tumors Predicted from Integrated Genomic Profiles
Source: PLoS One. 2011 Nov 3;6(11):e24709. doi: 10.1371/journal.pone.0024709 (PMC3207809; doi:10.1371/journal.pone.0024709)
Supplement: File S3 — Supporting information on microRNA expression data for “Time to recurrence and survival in serous ovarian tumors predicted from integrated genomic profiles”. (PDF) [file pone.0024709.s006.pdf]

Time to Recurrence and Survival in Serous Ovarian Tumors Predicted from Integrated Genomic Profiles

Parminder K. Mankoo, Ronglai Shen, Nikolaus Schultz, Douglas A. Levine and Chris Sander

microRNA expression

microRNA data was available for 527 samples. These intensities were $\log_2$ transformed and normalized using Robust Multiarray Averaging (RMA, AgiMicroRNA package in R).

Progression Free Survival: At λ=.858, we selected 81 microRNA features (Table ST8 [1]), cv.CPE = 0.704. Tertile stratification based on training data led to a p-value of 0.09 for the test data (Figures 3SA, 3SB).
Survival: At λ=.869, we selected 87 features (Table ST9 [2]), cv.CPE = 0.71. Tertile stratification based on training data led to a p-value of 0.09 for the test data (Figures 3SA, 3SB).

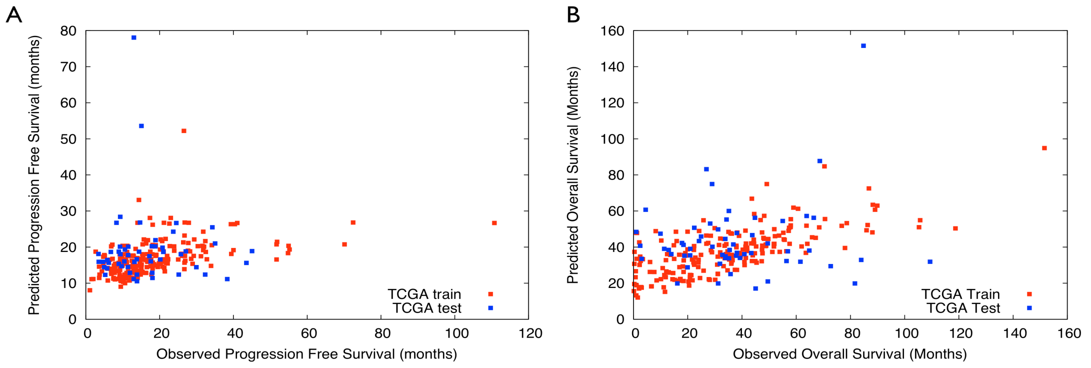

Figure 3SA: Predictions of median *time-to-event* using microRNA signatures: (A) Progression Free Survival and (B) Overall Survival; both for uncensored data.

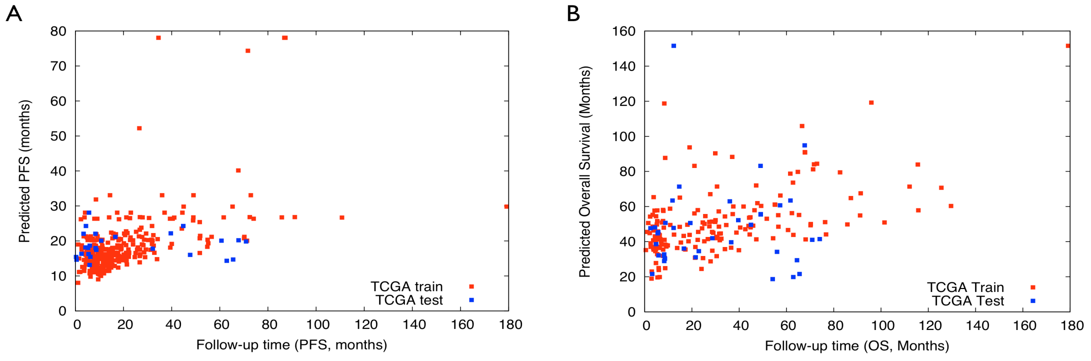

Figure 3SB: Median *time-to-event* predictions for censored data using microRNA signatures: (A) Progression Free Survival and (B) Overall Survival.

References:

[1] http://cbio.mskcc.org/~mankoo/ST8.txt

[2] http://cbio.mskcc.org/~mankoo/ST9.txt
