# Supplementary material for: Time to Recurrence and Survival in Serous Ovarian Tumors Predicted from Integrated Genomic Profiles
Source: PLoS One. 2011 Nov 3;6(11):e24709. doi: 10.1371/journal.pone.0024709 (PMC3207809; doi:10.1371/journal.pone.0024709)
Supplement: File S4 — Supporting information on copy number alteration data for “Time to recurrence and survival in serous ovarian tumors predicted from integrated genomic profiles”. (PDF) [file pone.0024709.s007.pdf]

Time to Recurrence and Survival in Serous Ovarian Tumors Predicted from Integrated Genomic Profiles

Parminder K. Mankoo, Ronglai Shen, Nikolaus Schultz, Douglas A. Levine and Chris Sander

Copy Number Alteration

Larger sample sizes in TCGA data compared to previous studies have made it possible to identify the copy number alterations (CNA) that correlate with outcome measures despite the heterogeneity of copy number alterations observed in SeOvCa. Gene specific calls from the GISTIC method [1] for 502 samples were used, generated for 31,324 gene isoforms in 502 samples. In the case of multi-isoform genes in 481 samples of interest, we selected the isoforms with the largest sum of the squares of the individual values. This procedure generated 20,871 features for 481 samples.

Progression Free Survival: At λ=.09 we selected 167 features (Table ST10 [2]), cv.CPE = 0.67, and the CPE for the training data was 0.68. Tertile stratification led to a p-value for the test data = 0.61, CPE.test = 0.67 (Figures 4SA, 4SB).

Survival: At λ=.211, we selected 278 features (Table ST11 [3]), CPE.test = 0.752. Tertile stratification led to the p-value for the test data = 0.18 (Figures 4SA, 4SB).

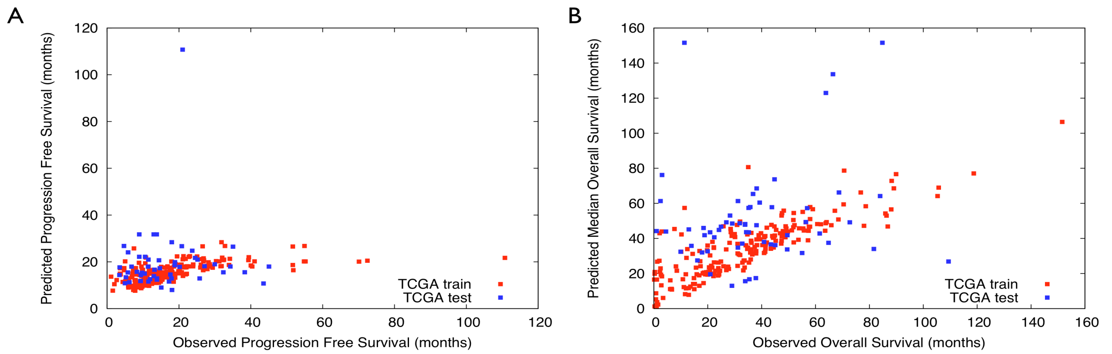

Figure 4SA: Time predictions using CNA signatures: (A) Progression Free Survival and (B) Overall Survival; both for uncensored data.

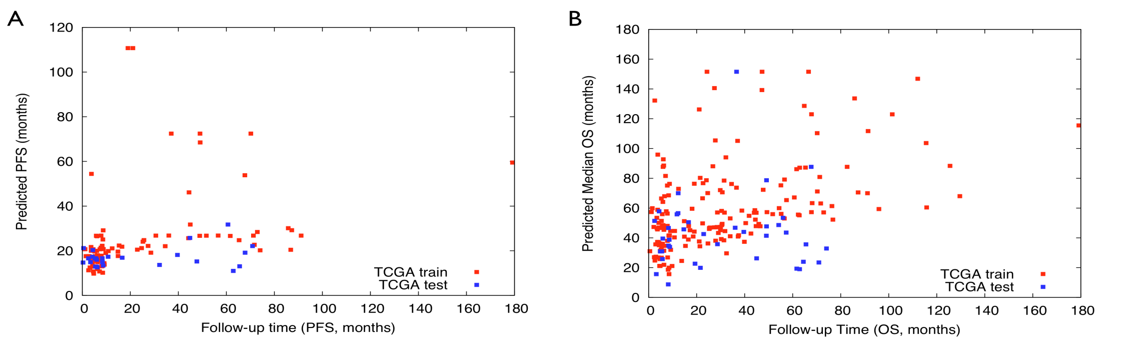

Figure 4SB: Time predictions using CNA signatures for censored data: (A) Progression Free Survival and (B) Overall Survival.

Copy number Alteration PFS signature: IPA analysis revealed Ubiquinone biosynthesis; Tight junction signaling; virus entry via endocytic pathways; CXCR4 signaling; Protein kinase A signaling; thrombin signaling; camp-mediated signaling and phospholipase signaling as the most statistically significant canonical pathways (Figure 4SC). The list of significant general biological functions include genetic disorder; cell death; cellular growth and proliferation; DNA replication, recombination & repair; cell cycle and cell signaling; drug metabolism and molecular transport; cancer and cellular response to therapeutics.

Copy number alteration OS Signature: The top list of canonical pathways from copy number survival signature include pancreatic cancer signaling; H1Fα signaling; IL-17 and IL-22 signaling; HMGB1 signaling; ovarian cancer signaling; colorectal cancer metastasis signaling; RAR activation; SAPK/JNK signaling; MYC mediated apoptosis signaling; TGF-β signaling; NF-κβ signaling; Wnt/β-catenin signaling; and p53 signaling and such (Figure 4SD).

The common pathways and functions emerging from both integrated and copy number data in serous ovarian cancer are DNA replication, recombination & repair; cellular growth and proliferation, drug and molecular transport and metabolism, cell-to-cell signaling and interaction; and cellular growth and proliferation.

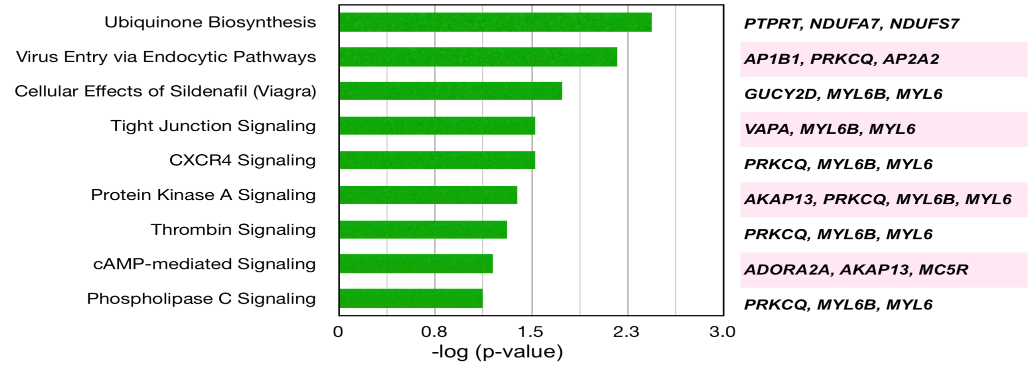

Figure 4SC: Canonical pathway analysis of 167 PFS copy number features: Top functions with p-value cut-off < 0.1 are depicted.

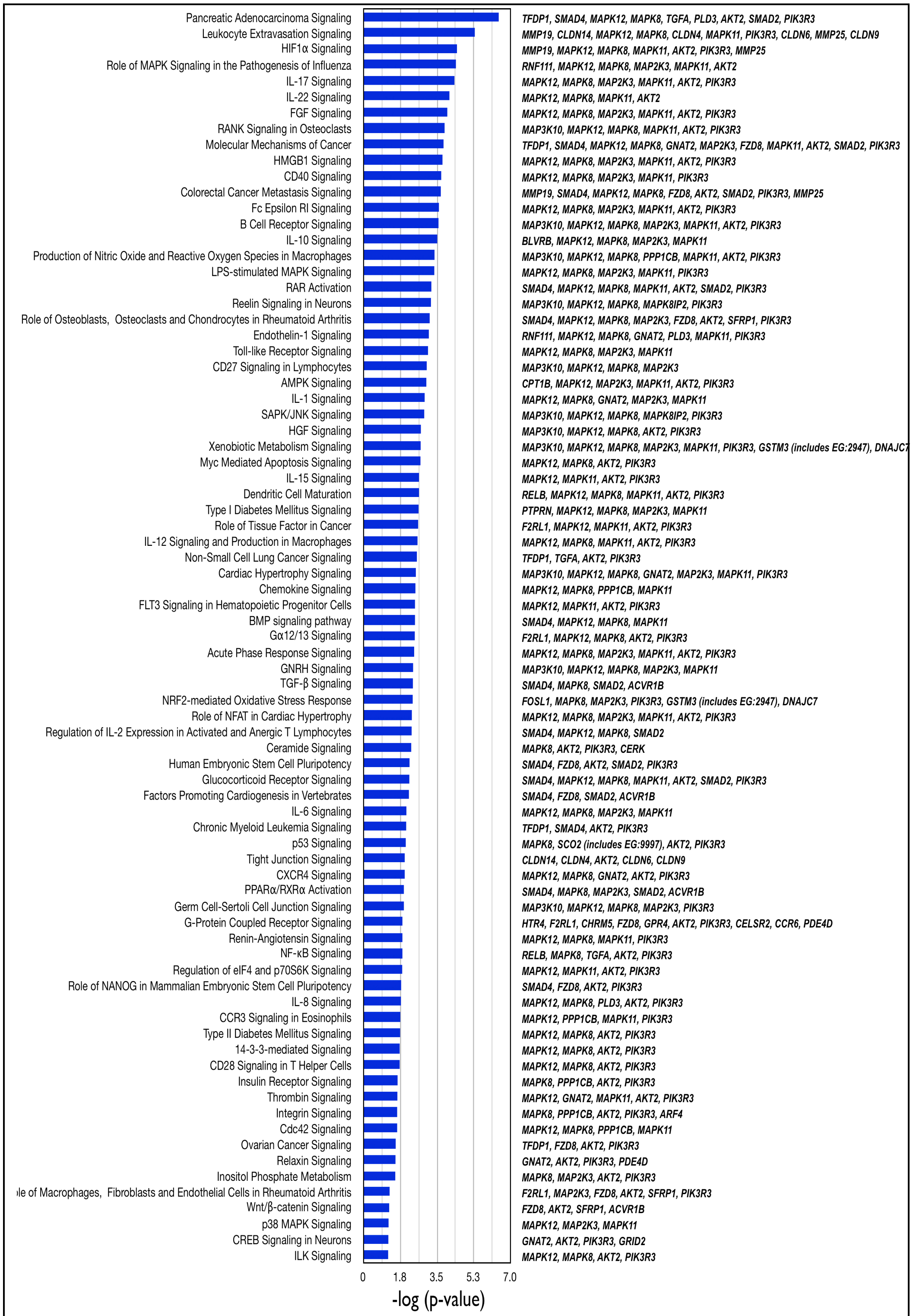

Figure 4SD: Canonical pathway analysis of 278 OS copy number features: Top functions with p-value cut-off < 0.1 are depicted.

References:

1. Beroukhim R, Mermel CH, Porter D, Wei G, Raychaudhuri S, et al. The landscape of somatic copy-number alteration across human cancers. Nature 463: 899-905.
2. http://cbio.mskcc.org/~mankoo/ST10.txt
3. http://cbio.mskcc.org/~mankoo/ST11.txt
