# Supplementary material for: Time to Recurrence and Survival in Serous Ovarian Tumors Predicted from Integrated Genomic Profiles
Source: PLoS One. 2011 Nov 3;6(11):e24709. doi: 10.1371/journal.pone.0024709 (PMC3207809; doi:10.1371/journal.pone.0024709)
Supplement: File S5 — Supporting information on integrated data for “Time to recurrence and survival in serous ovarian tumors predicted from integrated genomic profiles”. (PDF) [file pone.0024709.s008.pdf]

# Time to Recurrence and Survival in Serous Ovarian Tumors Predicted from Integrated Genomic Profiles

*Parminder K. Mankoo, Ronglai Shen, Nikolaus Schultz, Douglas A. Levine and Chris Sander*

## Integrated data

Vector space integration of four different datatypes was performed as explained in the Materials and Methods section in the main text. Here, we summarize the results of correlation analysis in the integration procedure for each outcome measure (PFS and OS).

*Progression Free Survival*: We began by computing the Spearman rank correlations of the mRNA datasets with other datasets as described in Figure 2 in the main text, which resulted in 432 microRNA features (cut-off = coef < -0.3), 4563 DNA methylation features (cut-off = coef < -0.05), 6243 (cut-off = coef > 0.3) copy-number features, and 9198 mRNA features. At λ=.848, we selected 156 features (Table ST12 [1]) most associated with time to progression and se(coef) of all coefficients < 1.0. The cross-validation estimate of the concordance probability estimate (cv.CPE) was 0.738, which indicated very good discriminatory and predictive power of this signature. There are 85 mRNA features, 47 DNA-methylation features, 18 copy-number feature, and 6 microRNA features (Figures 2 and 5SA).

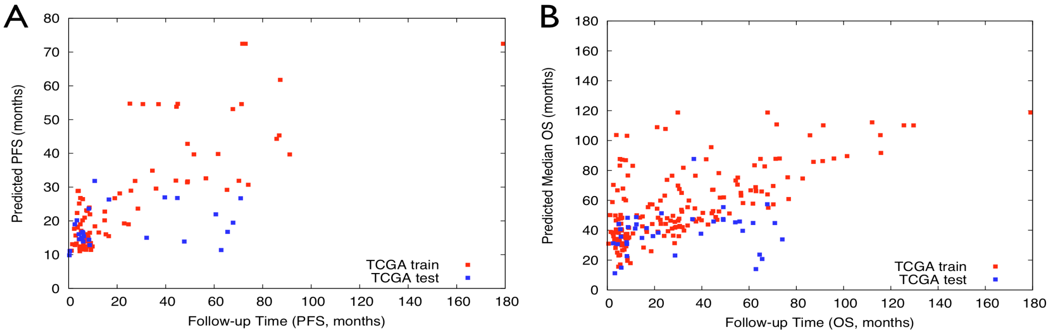


**Figure 5SA: Median *time-to-event* prediction using integrated signatures (censored data):** (A) Prediction of median *time-to-event* (PFS; censored data); (B) Prediction of median *time-to-event* (OS; censored data).

*Survival*: Integrated feature extraction associated with overall survival and four datatype correlation analysis, led to 432 microRNA features (cut-off = coef < -0.3), 4641 DNA-methylation features (cut-off = coef < -0.05), 6292 (cut-off = coef > 0.3) copy-number features, and 9091 mRNA features. At λ=.869, we selected 182 features (Table ST13 [2]) most associated with overall survival and se(coef) of all coefficients < 1.0. There are 102 mRNA features, 40 methylation features, 30 copy-number features, and 10 microRNA features (Figures 3 and 5SA).

*Integrated Overall Survival:* IPA analysis to identify the top canonical pathways revealed G protein Signaling, Phospholipase signaling, Role of Tissue factor in cancer, Androgen signaling and such. IPA investigation of general biological functions includes cellular development, cellular growth and proliferation, reproductive system development, lipid metabolism, drug metabolism, cancer; DNA replication, recombination and repair, etc. (Figure 5SB).

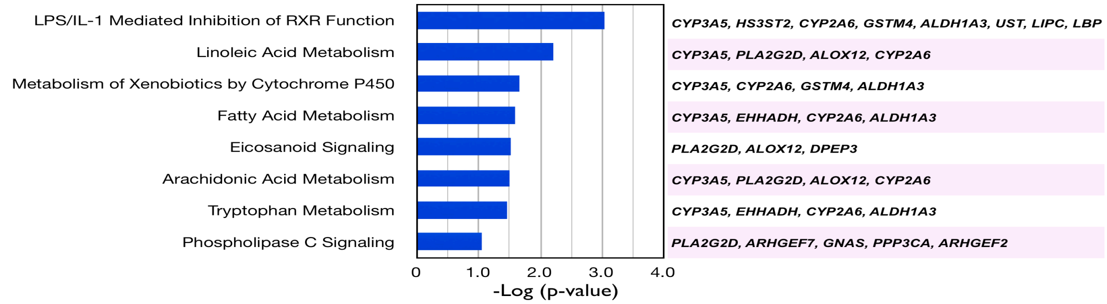


**Figure 5SB: Functional analysis of 182 survival features:** Top canonical pathways with p-value cut-off < 0.1 and inclusive of ≥ 3 genes are depicted.

## Software and Hardware:

All calculations were performed on a Dell PowerEdge R900 cluster. R version 2.9.2 (X86_64, linux-gnu) and Perl programming language were used for writing scripts. The R libraries 'glmpath' and 'cpe' were used for coxpath and concordance probability calculations, respectively.

## References:

[1] http://cbio.mskcc.org/~mankoo/ST12.txt

[2] http://cbio.mskcc.org/~mankoo/ST13.txt
